# Basophil Extracellular Traps in Immunity and Disease

**DOI:** 10.3390/cancers18183006

**Published:** 2026-09-16

**Authors:** Bojan Stojanovic, Ivana Milivojcevic Bevc, Milica Dimitrijevic Stojanovic, Bojan Milosevic, Darko Laketic, Nenad Zornic, Vesna Vulovic, Verica Vukicevic, Danijela Bazic Sretenovic, Sladjan Petrovic, Sanja Knezevic, Jelena Nesic, Stevan Eric, Goran Marjanovic, Vojislav Cupurdija, Aleksandar Matic

**Affiliations:** 1 Department of Surgery, Faculty of Medical Sciences, University of Kragujevac, 34000 Kragujevac, Serbia; 2 Center for Molecular Medicine and Stem Cell Research, Faculty of Medical Sciences, University of Kragujevac, 34000 Kragujevac, Serbia; 3 Institute for Emergency Medicine Belgrade, 11000 Belgrade, Serbia; 4 Department of Pathology, Faculty of Medical Sciences, University of Kragujevac, 34000 Kragujevac, Serbia; 5 Institute of Anatomy, Faculty of Medicine, University of Belgrade, 11000 Belgrade, Serbia; 6 Department of Surgery, University Hospital Medical Center Bežanijska Kosa, 11000 Belgrade, Serbia; 7 Department of Internal Medicine, Faculty of Medical Sciences, University of Kragujevac, 34000 Kragujevac, Serbia; 8 Department of Surgery, Health Center Prokuplje, 18400 Prokuplje, Serbia; 9 Department of Pediatrics, Faculty of Medical Sciences, University of Kragujevac, 34000 Kragujevac, Serbia; 10 General Hospital Moj Lab, 81000 Podgorica, Montenegro

**Keywords:** basophils, basophil extracellular traps, mitochondrial DNA, mitochondrial reactive oxygen species, IL-3, FcεRI, C5a, antimicrobial immunity, inflammation, cancer

## Abstract

Basophil extracellular traps (BETs) represent a recently recognized extracellular effector mechanism whose biology remains far less defined than that of neutrophil extracellular traps. This review critically examines the terminology, molecular composition, signaling requirements, and disease relevance of BET formation. Current evidence supports a rapid, predominantly non-lytic process in which mitochondrial reactive oxygen species promote the externalization of mitochondrial DNA, particularly after IL-3 priming followed by FcεRI or C5a receptor activation. The resulting networks contain mtDNA associated with basophil granule proteins, including basogranulin in humans and mMCP-8 in mice, although their complete proteomic composition and route of cellular export remain unresolved. Functional studies indicate that BETs can immobilize *Escherichia coli* and *Staphylococcus aureus* and contribute to extracellular bacterial killing, while in vivo observations place BET release in helminth-associated inflammation and several inflammatory dermatoses. A small cervical cancer study reported circulating BET-like structures during anticancer treatment, although their basophil origin and molecular composition were not comprehensively confirmed and their biological significance remains unknown. By separating direct basophil evidence from mechanisms inferred from other trap-forming cells, this review defines the current experimental boundaries of the field and highlights priorities for mechanistic and translational investigation.

## Simple Summary

Basophils are rare immune cells best known for their role in allergic reactions, but they can also release web-like structures made mainly of mitochondrial DNA and cell proteins. These structures, called basophil extracellular traps, may help capture and kill bacteria, yet they may also contribute to inflammation when they accumulate or persist in tissues. Because this field is still new, many basic questions remain unanswered, including how these traps are formed, how their components leave the cell, and whether they are protective or harmful in different diseases. This review brings together current evidence on their formation, composition, antimicrobial effects, and possible involvement in inflammatory skin disorders, parasite-related inflammation, and cancer. By clearly separating findings demonstrated in basophils from mechanisms inferred from other immune cells, the review identifies major knowledge gaps and provides a framework for future laboratory and clinical studies.

## 1. Introduction

Basophils are rare circulating granulocytes best known for their involvement in immunoglobulin E (IgE)-mediated allergy and type 2 immune responses, although their functions also include antimicrobial defense, tissue inflammation, regulation of other immune cells, and tumor-associated immunity [1]. Beyond releasing soluble mediators and granule contents, activated basophils can form basophil extracellular traps (BETs), extracellular networks composed mainly of mitochondrial DNA (mtDNA) and basophil-derived proteins [2]. Current evidence suggests that BETs can be released rapidly without immediate cell lysis and may be triggered by allergic, microbial, complement-derived, cytokine-mediated, and sterile inflammatory stimuli [3,4]. BETs have been associated with extracellular bacterial control and have been observed in experimental helminth infection, inflammatory skin lesions, and, on the basis of still limited evidence, in patients with cancer [3,5,6]. However, the intracellular mechanisms governing mtDNA mobilization, the complete molecular composition of these structures, and their protective or pathological effects remain incompletely defined. This review therefore integrates current knowledge on BET formation, regulation, structure, and biological functions, while clearly distinguishing experimentally established findings from mechanisms inferred from other extracellular-trap systems and identifying the principal questions that should guide future research.

## 2. Basophils: Development, Activation, and Immune Functions

Basophils were first identified as a distinct blood granulocyte more than 140 years ago, when Paul Ehrlich used basic aniline dyes to demonstrate their characteristic metachromatic staining [7]. They are now recognized as bone marrow-derived granulocytes that normally represent less than 1% of circulating leukocytes [1]. Their cytoplasm contains numerous intensely staining granules rich in histamine, basogranulin, and various preformed inflammatory mediators [8]. Because basophils have a relatively short lifespan in the circulation, they are continuously replaced by cells arising from myeloid progenitors in the bone marrow [7]. Most basophils circulate in the bloodstream, although distinct basophil populations have also been identified in tissues. Allergic reactions, infections, helminth exposure, and other inflammatory stimuli can promote their migration into affected tissues [8].

Human and mouse basophils express the high-affinity IgE receptor, Fc epsilon receptor I (FcεRI). Cross-linking of FcεRI rapidly activates these cells, triggers granule release, and stimulates the production of additional mediators, which explains their well-established role in immediate hypersensitivity and anaphylaxis [9]. Basophils can also respond independently of IgE [9]. Microbial products can activate them through pattern-recognition receptors such as Toll-like receptor 2 (TLR2) and Toll-like receptor 4 (TLR4), whereas proteases derived from allergens or parasites and complement component 5a (C5a) provide additional activating signals [1,9]. IL-3 influences mature basophils by extending their survival and increasing their responsiveness to subsequent receptor-dependent and innate stimuli, although IL-3 alone does not always induce a complete effector response [10,11]. Basophil characteristics are also shaped during development. In mice, basophils that develop in the presence of IL-3 differ transcriptionally and functionally from those influenced by thymic stromal lymphopoietin (TSLP), suggesting that basophils do not constitute a single uniform population [12].

**Figure 1:**
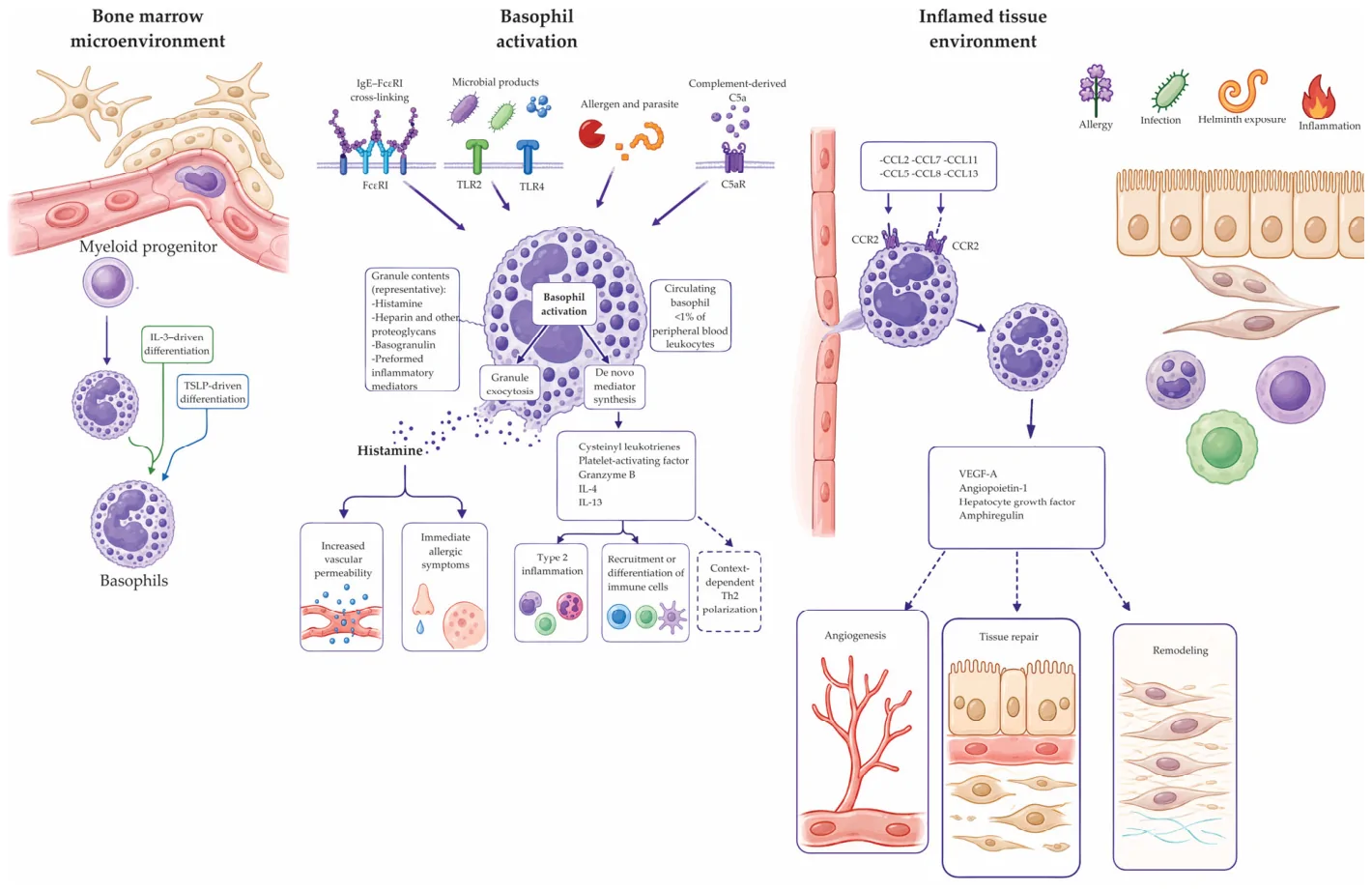
Basophil development, activation, and transition from circulating to tissue effector cells. Basophils arise from bone marrow myeloid progenitors under cytokine-dependent differentiation programs involving IL-3 and TSLP and subsequently enter the circulation as a low-abundance granulocyte population. Once activated through FcεRI or innate receptors by allergens, microbial products, complement-derived signals, cytokines, chemokines, or other inflammatory stimuli, basophils release preformed and newly synthesized mediators, including histamine, lipid mediators, cytokines, chemokines, and proteolytic enzymes. These products contribute to immediate vascular and allergic responses, promote type 2 inflammation, and influence the recruitment and activity of neighboring immune cells. During sustained inflammation, chemokine-receptor signaling, particularly through CCR2 and CCR3, promotes basophil recruitment from the circulation into affected tissues, where their functional profile is shaped by the local microenvironment. Tissue-associated basophils may then participate in allergic and infectious inflammation and contribute to angiogenesis, tissue repair, and remodeling. The figure therefore summarizes basophil biology as a continuous sequence from bone marrow development and systemic circulation to stimulus-dependent activation and context-specific functions within inflamed tissues.

Activated basophils release stored granule contents together with newly synthesized cytokines, chemokines, lipid mediators, and proteolytic enzymes [13]. These mediators include histamine, cysteinyl leukotrienes (CysLTs), platelet-activating factor (PAF), granzyme B (GzmB), interleukin-4 (IL-4), and interleukin-13 (IL-13) [13]. Collectively, they increase vascular permeability, contribute to acute allergic symptoms and type 2 inflammation, and influence the recruitment and differentiation of other immune cells [14]. Experimental studies also suggest that basophils can promote T helper 2 (Th2) cell polarization and function as antigen-presenting cells, although the physiological importance of these activities appears to depend on the experimental model [8,15,16]. Basophils may also contribute to longer-term tissue responses by producing vascular endothelial growth factor A (VEGF-A), angiopoietin-1 (Ang-1), hepatocyte growth factor (HGF), and amphiregulin (AREG), which may support angiogenesis, tissue repair, and remodeling [8,17]. Their migration into inflamed tissues is partly regulated by C-C chemokine receptor 2 (CCR2) and C-C chemokine receptor 3 (CCR3), together with chemokines such as C-C motif chemokine ligand 2 (CCL2), C-C motif chemokine ligand 5 (CCL5), C-C motif chemokine ligand 7 (CCL7), C-C motif chemokine ligand 8 (CCL8), C-C motif chemokine ligand 11 (CCL11), and C-C motif chemokine ligand 13 (CCL13) [18]. Basophil characteristics vary between species and are influenced by developmental conditions, tissue localization, and activation state [7]. Basophils that migrate into tissues may therefore acquire features distinct from those of circulating cells, which themselves comprise phenotypically diverse subsets [7,19]. These differences should be considered when interpreting experimental findings. In complex inflammatory infiltrates, reliable identification generally requires basophil-specific molecular markers because cellular morphology alone is often insufficient [20]. Taken together, basophil biology can be viewed as a continuum extending from bone marrow differentiation and circulation to stimulus-dependent activation, chemokine-guided tissue recruitment, and context-specific effector functions within inflamed tissues (Figure 1).

## 3. Extracellular Trap Formation and ETosis

The term extracellular trap-associated cell death (ETosis) was initially introduced to describe a distinct form of cell death in which chromatin undergoes extensive decondensation, the nuclear envelope disintegrates, and nuclear material becomes associated with granular and cytoplasmic proteins before rupture of the plasma membrane releases extracellular traps (ETs) [21]. Subsequent studies demonstrated, however, that DNA can also be released without cell lysis or an immediate loss of viability [22]. ET formation is therefore a broader concept that encompasses both lytic, or suicidal, ETosis and vital pathways in which the cell remains temporarily viable [22,23]. During lytic ETosis, the nucleus progressively disintegrates, the plasma membrane ruptures, and cellular viability is irreversibly lost [24]. During vital ET formation, DNA is released in a more controlled manner without immediate disruption of the plasma membrane, allowing at least some cellular functions to be preserved [22].

ETs were first described in neutrophils as antimicrobial structures that immobilize microorganisms and retain toxic mediators at sites of infection [25,26]. Similar DNA-based structures were subsequently identified in eosinophils, basophils, mast cells, macrophages, plasmacytoid dendritic cells (pDCs), and certain lymphocyte populations, giving rise to terminology based on the cell type from which they originate [2,27]. These findings also demonstrated that ETs do not represent a single uniform entity. Their composition and mode of release vary according to the cell of origin, activating stimulus, degree of cellular priming, metabolic conditions, and surrounding tissue environment [28]. ETs can also be classified according to whether their DNA scaffold is derived from nuclear DNA (nDNA) or mtDNA [2,29]. nDNA is most commonly released during lytic chromatin extrusion, although vesicular export without immediate cell death has been demonstrated in some neutrophil models [29]. By contrast, mtDNA can be released rapidly through pathways associated with mitochondrial reactive oxygen species (ROS), while nuclear architecture and early cellular viability remain preserved [30]. However, the origin of the DNA does not itself determine whether ET formation is lytic or non-lytic, because both nDNA and mtDNA may be externalized under different cellular conditions [2]. DNA origin and cell viability should therefore be evaluated as separate characteristics of ET formation [28].

### 3.1. Classification of Extracellular Trap-Forming Pathways and Their Relevance to BET Formation

Because most mechanistic models of ET formation were established in neutrophils, they provide a useful framework for BET biology; however, it remains important to distinguish mechanisms demonstrated directly in basophils from those inferred from other extracellular trap systems. Suicidal, or lytic, ETosis is a form of ET release that culminates in irreversible loss of cell viability and rupture of the plasma membrane [28]. It has been studied most extensively in neutrophils, in which classical experimental models usually develop over one to four hours, although similar lytic responses have also been reported in other immune cells under certain experimental or pathological conditions [26,29]. Depending on the cell type and activating stimulus, lytic ET formation may be induced by microorganisms, immune complexes, autoantibodies, inflammatory cytokines, oxidants, crystals, or potent pharmacological activators [28]. In the classical neutrophil model induced by phorbol 12-myristate 13-acetate (PMA), activation of protein kinase C (PKC) and the rapidly accelerated fibrosarcoma–mitogen-activated protein kinase kinase–extracellular signal-regulated kinase (Raf–MEK–ERK) signaling pathway stimulates nicotinamide adenine dinucleotide phosphate (NADPH) oxidase and increases ROS production [29,31]. Neutrophil elastase (NE) and myeloperoxidase (MPO) are subsequently released from granules and translocate to the nucleus, where they contribute to histone degradation and chromatin relaxation [32]. In calcium-dependent pathways, peptidylarginine deiminase 4 (PAD4) may also promote chromatin decondensation by citrullinating histones, thereby reducing their positive charge and weakening their interaction with DNA [24,28,29]. As the process progresses, the nucleus loses its lobulated morphology, and the nuclear envelope disintegrates, allowing decondensed chromatin to mix with cytoplasmic and granule proteins [29]. The resulting DNA–protein material is eventually released following rupture of the plasma membrane. In some lytic pathways, gasdermin D (GSDMD) may facilitate this process by forming pores in granule and plasma membranes, thereby promoting the redistribution of granule enzymes and the terminal loss of membrane integrity [24,29]. However, this sequence does not apply to every form of lytic ET release. The involvement of NADPH oxidase, PAD4, granule proteases, gasdermins, and nuclear chromatin extrusion varies among stimuli and cell types [33]. Suicidal ETosis is therefore best identified by its final morphological and functional characteristics, including irreversible nuclear breakdown, loss of plasma membrane integrity, and extracellular release of DNA associated with cellular proteins, rather than by the presence of any single signaling pathway [34].

Vital ET formation, more cautiously described as non-lytic ET release, involves the externalization of DNA without immediate rupture of the plasma membrane or clear evidence of irreversible cell death [35,36]. This process has been studied most extensively in neutrophils and generally occurs more rapidly than classical suicidal ETosis [37]. In some experimental models, cells temporarily retain functions such as migration, chemotaxis, and phagocytosis [37]. nDNA may be externalized through budding of the nuclear envelope, vesicular transport across the cytoplasm, and subsequent fusion of DNA-containing vesicles with the plasma membrane [38]. In other non-lytic pathways, the released DNA originates from mitochondria. The principal feature distinguishing these responses from lytic ETosis is therefore the preservation of cellular integrity rather than the nuclear or mitochondrial origin of the extracellular DNA [39]. Rapid nDNA release has been observed during severe infections, including platelet-dependent responses in sepsis. In these models, TLR4-activated platelets stimulate neutrophils to form ETs within minutes, whereas membrane-impermeable dyes show limited entry into the cells [40]. *Staphylococcus aureus* can also induce ET release within approximately 5–60 min through a TLR2-dependent pathway that appears to occur independently of conventional NADPH oxidase-derived ROS production [38]. These responses differ from the slower lytic ET formation induced by PMA [29]. However, rapid release and temporary preservation of plasma membrane integrity do not by themselves demonstrate that the cell remains viable or fully functional over time [39]. Cellular viability and retained functions should therefore be evaluated using several complementary methods. The underlying mechanisms also differ among immune cell types. Vesicular release of nDNA has been described mainly in neutrophils, whereas rapid non-lytic release of mtDNA has been reported in eosinophils and basophils [3,41]. The term vital should therefore be used cautiously when the available evidence demonstrates only the absence of immediate lysis. DNA origin, route of release, signaling pathways, retained cellular functions, and subsequent cell fate should be evaluated separately for each cell type and stimulus. Among the pathways described above, this rapid mtDNA-associated response most closely resembles the BET phenotype reported in primary human basophils. However, preserved membrane integrity at the time of DNA release only supports the absence of immediate lysis; it does not show that the basophils remain viable and functionally active thereafter. For this reason, the available evidence supports describing BET release as non-lytic rather than as a definitively vital process.

Mitochondrial DNA-based extracellular trap formation represents a rapid trap-releasing pathway in which mitochondrial DNA, rather than nuclear chromatin, provides the principal extracellular scaffold [30]. It has been described in several granulocyte populations and commonly occurs without extensive nuclear disintegration, early plasma membrane rupture, or immediate loss of cellular viability [42]. Nevertheless, mitochondrial origin of the released DNA should not be taken as sufficient evidence of a vital process, because cellular survival and functional preservation must be demonstrated independently [39]. This pathway was first described in neutrophils, in which priming with granulocyte–macrophage colony-stimulating factor (GM-CSF) followed by stimulation with lipopolysaccharide (LPS) or C5a induced mtDNA release within approximately 15–20 min [30]. *Staphylococcus aureus* can also induce mitochondrial ET formation, with oxidative signals generated by mitochondrial respiratory chain complex III contributing to the response [43]. Mitochondrial reactive oxygen species (mtROS) therefore appear to regulate mtDNA mobilization and release, although the precise respiratory-chain source and redox-sensitive molecular targets involved may vary depending on the stimulus [30]. In neutrophils, small-conductance calcium-activated potassium (SK) channels have also been associated with mtDNA release, pointing to a possible interplay between calcium signaling, ion-channel activity, and mitochondrial ROS production [44]. Other mechanisms have thus far been identified only in specific disease models. In aged tumor-associated neutrophils (TANs), sirtuin 1 (SIRT1)-related signaling has been associated with opening of the mitochondrial permeability transition pore (mPTP), whereas optic atrophy 1 (OPA1) appears to support the mitochondrial and microtubular changes required for mtDNA transport [45]. These findings should not yet be extrapolated beyond the experimental systems in which they were observed. These neutrophil studies provide examples of how mitochondrial activation may result in extracellular mtDNA release, but the same mechanisms have not been established for BET formation. In particular, SK-channel signaling, SIRT1-dependent mPTP opening, OPA1-mediated mitochondrial remodeling, and related processes have not yet been demonstrated in basophils. Similar mtDNA-based ETs have been reported in eosinophils and in primed and activated basophils, in which the released DNA subsequently associates with granule-derived proteins [3,41]. In basophils, mtDNA release depends on mtROS production but not on the conventional NADPH oxidase 2 (NOX2)-dependent respiratory burst [3,30]. Mitochondria may therefore provide both the oxidative signals that initiate ET formation and the DNA that forms the extracellular network [30]. However, it remains unclear how mtDNA crosses the mitochondrial membranes, moves through the cytoplasm, and ultimately exits the cell, and these steps are likely to differ among cell types and activating stimuli. Taken together, BETs share the basic structural features of other extracellular traps, consisting of extracellular DNA associated with proteins released from the producing cell. However, the mechanism most clearly described in basophils differs in several respects from classical NET formation. The extracellular DNA is predominantly mitochondrial in origin, its release occurs rapidly and without obvious disruption of the nucleus, oxidant production is linked mainly to mitochondrial activity rather than to conventional NOX2-dependent pathways, and the released DNA is associated with basophil granule proteins. None of these features is completely restricted to basophils, since rapid extracellular release of mtDNA has also been reported in eosinophils and in some neutrophil models. BET formation is therefore better regarded as a basophil-specific variation in extracellular trap release, with features shared with other ET-forming cells, rather than as a wholly distinct process or a direct counterpart of NET formation.

### 3.2. Protective and Pathological Effects of Extracellular Traps

The biological effects of extracellular DNA are influenced by the amount released, its local accumulation, the proteins carried within the extracellular material, and the efficiency of its removal [34]. Most of what is currently known about the protective and pathological consequences of extracellular trap formation comes from studies of NETs and, to a lesser extent, traps released by other immune cells. In extracellular traps, DNA forms a fibrous network that captures microorganisms, restricts their spread, and retains antimicrobial proteins and enzymes near the trapped pathogens, thereby facilitating their phagocytic clearance [46]. These traps may also contain crystals, cellular debris, and necrotic material, reducing their immediate contact with viable cells [47,48]. However, these protective effects may be lost when traps are produced in excess or remain in tissues for prolonged periods [34]. Dense trap deposits can block glandular ducts, promote microbial biofilm formation, and expose surrounding tissues to histones, proteases, and other harmful components [48,49,50]. Persistent DNA and protein complexes may also reveal autoantigens, maintain inflammatory signaling, and contribute to microvascular obstruction, thrombosis, fibrosis, and chronic tissue damage [51]. The effect of extracellular DNA therefore depends on the balance between its ability to contain harmful material locally and the inflammatory, immunogenic, and physical damage that may result when traps accumulate. For BETs specifically, direct evidence currently supports microbial entrapment and antibacterial activity more strongly than the broader inflammatory, vascular, or tissue-damaging effects described for other ET systems.

#### 3.2.1. Immune Activation by Extracellular DNA

Beyond serving as the structural scaffold of ETs, extracellular DNA can also act as a proinflammatory stimulus, particularly when it remains associated with histones, antimicrobial peptides (AMPs), or other proteins released by the producing cell [52]. These molecular interactions may protect DNA from nuclease-mediated degradation, prolong its persistence within tissues, and facilitate its uptake by immune cells, where it can be recognized by intracellular nucleic acid sensors [52,53]. pDCs are particularly responsive to DNA-containing complexes, which can activate endosomal Toll-like receptor 9 (TLR9) and stimulate the production of type I interferons (IFN-I) [53]. ET-derived material may also activate monocytes, macrophages, and the complement system, thereby extending the inflammatory response beyond the cell population that originally released the traps [54]. When exposure persists, extracellular DNA together with structurally altered or oxidized proteins may weaken immune tolerance to self-antigens and provide a sustained source of antigenic material for autoreactive B cells, promoting autoantibody production and the formation of immune complexes (ICs) [34,52]. These ICs can subsequently induce additional ET release, establishing a self-reinforcing cycle of extracellular DNA accumulation, innate immune activation, autoantibody responses, and tissue injury [55,56]. Oxidized mtDNA appears to be particularly immunogenic and may further amplify IFN-I-driven inflammation [57].

#### 3.2.2. Extracellular Trap-Mediated Endothelial and Epithelial Injury

ETs can damage endothelial and epithelial barriers through the combined effects of their molecular components [50,58]. Histones bound to extracellular DNA can interact with cell membranes and directly compromise their integrity, whereas signaling through TLR2, TLR4, and myeloid differentiation primary response 88 (MyD88) can increase the production of inflammatory mediators [59]. Proteases carried by ETs may further damage barrier tissues by degrading extracellular matrix proteins, intercellular junctions, and protective molecules on epithelial and endothelial surfaces [59,60]. Within blood vessels, ETs can adhere to endothelial cells, promote a proinflammatory state, and recruit leukocytes and platelets [59]. Endothelial injury may then induce additional ET release, further aggravating vascular inflammation and microcirculatory disturbances [61,62]. Similar effects have been described in renal tubular cells and other epithelial tissues, where they may contribute to local inflammation, loss of junctional integrity, impaired barrier function, cellular dysfunction, and, in more severe cases, cell death [58,61]. Tissue injury therefore results not from extracellular DNA alone but from prolonged exposure to histones, proteases, and other DNA-associated proteins, together with the inflammatory responses sustained by these components.

#### 3.2.3. Antimicrobial Functions and Microbial Evasion

ETs support innate defense by confining microorganisms within DNA-based networks and restricting their movement through infected tissues [34,63]. This physical containment may remain beneficial even when immediate microbial killing is incomplete because it delays dissemination and provides time for the recruitment and activation of additional immune cells [46,64]. The chromatin network also brings trapped pathogens into close contact with proteases, peroxidases, histones, and other antimicrobial proteins, thereby increasing their exposure to extracellular antimicrobial activity [25]. However, microorganisms can weaken this protective effect through several escape mechanisms. Pathogen-derived deoxyribonucleases (DNases) can degrade the DNA network, release captured microorganisms, and reduce their exposure to ET-associated proteins [65]. Changes in microbial surface composition or electrical charge may also weaken interactions with extracellular DNA and facilitate escape [66]. ET formation should therefore be regarded as one component of host defense rather than an invariably effective antimicrobial response. Its effectiveness depends on the stability of the DNA network, the activity of the proteins associated with it, and the ability of the pathogen to resist entrapment or dismantle the trap.

## 4. Basophil Extracellular Traps

In addition to releasing soluble mediators, activated basophils can form extracellular DNA–protein networks known as basophil extracellular traps [3]. These structures are composed mainly of mitochondrial DNA and can capture microorganisms while keeping basophil granule proteins close to their surface [5]. This suggests that BET formation is an active part of innate defense rather than simply a by-product of nonspecific cell damage [5]. The effects of BETs probably depend on how extensively they are formed and how long they remain in tissues. Brief and controlled release may help contain microorganisms, whereas prolonged accumulation of extracellular mtDNA and basophil-derived proteins may sustain local immune activation and contribute to tissue inflammation. Current evidence supports an antimicrobial role for BETs more clearly than a direct contribution to disease [5]. It is still unclear which receptors recognize BET-derived material, which signaling pathways are subsequently activated, and how these responses affect surrounding immune cells and tissues.

### 4.1. Inducers of Basophil Extracellular Trap Formation

BETs can form following activation through FcεRI, as well as in response to cytokines, chemokines, complement components, lipid mediators, and microbial products recognized by receptors such as Toll-like receptors (TLRs) [2,3]. In several studies, extracellular DNA appeared within minutes of stimulation, indicating that BET release can occur rapidly after basophil activation [3]. The broad range of reported triggers suggests that BET formation is not restricted to a single allergic, infectious, or inflammatory context but may be initiated through several receptor-dependent pathways. However, it remains unclear to what extent this response is shaped by stimulus intensity, prior cellular priming, or the combined effects of multiple signals.

The requirement for IL-3 priming depends on the stimulus that induces BET formation [3]. In human peripheral blood basophils, FcεRI cross-linking, C5a, CCL11, and TSLP induced extracellular DNA release only after prior exposure to IL-3 [3,23]. Consistent with this finding, combined stimulation with IL-3 and C5a produced stronger BET-associated antibacterial activity than bacterial stimulation alone, suggesting that priming and activating signals can act cooperatively to enhance the response [5]. In contrast, N-formyl-methionyl-leucyl-phenylalanine (fMLF), PAF, LPS, and lipoteichoic acid (LTA) triggered extracellular DNA release without prior IL-3 exposure [3]. These findings indicate that some signaling pathways require cellular priming, whereas others can initiate BET formation directly. Previous exposure to cytokines may therefore shape the magnitude and characteristics of basophil responses to subsequent activating signals [10].

**Figure 2:**
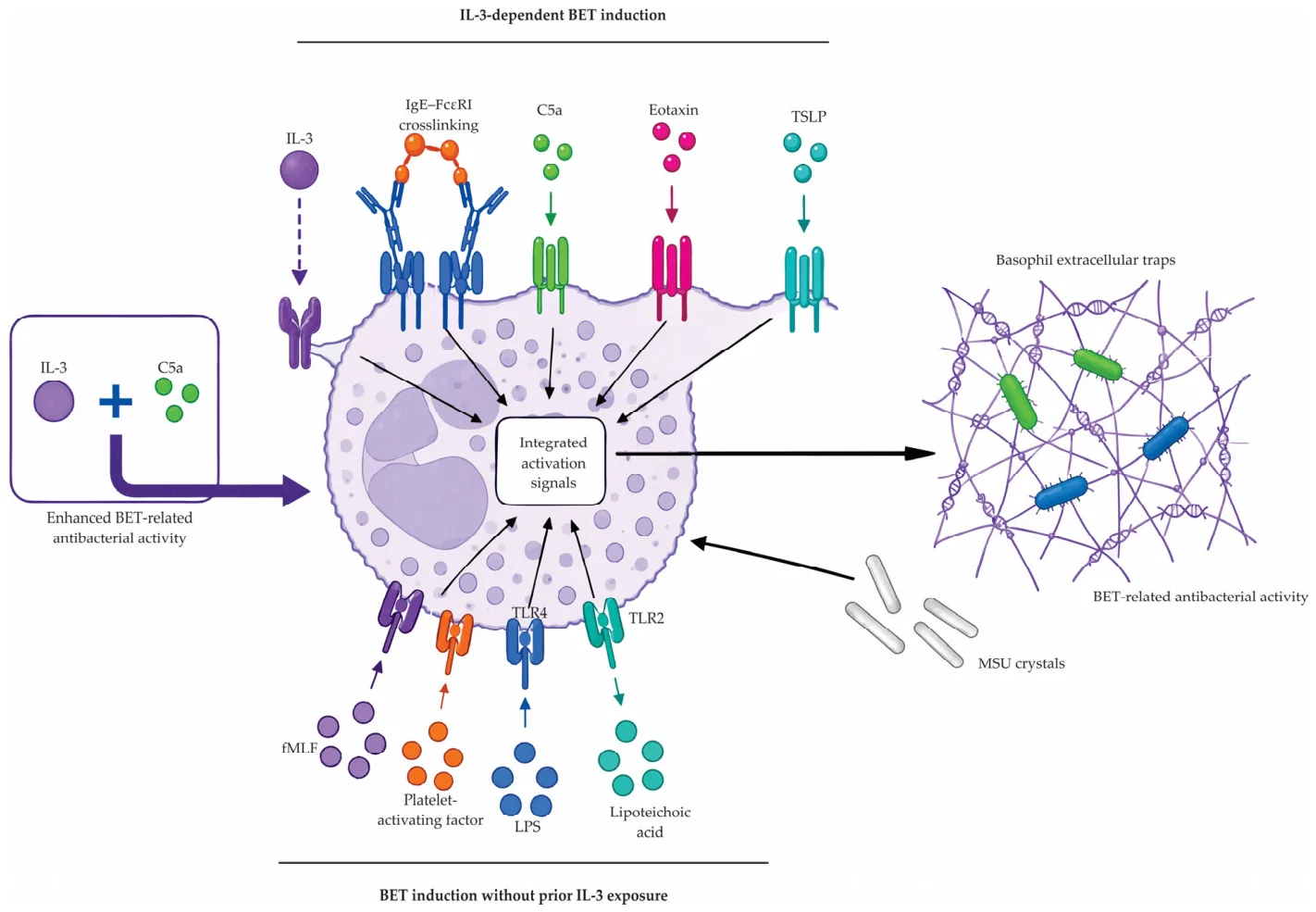
Stimulus-dependent induction and antibacterial activity of basophil extracellular traps. FcεRI cross-linking, C5a, eotaxin/CCL11, and TSLP induce BET formation after IL-3 priming, whereas fMLF, PAF, LPS, and LTA can act without prior IL-3 exposure. MSU crystals provide an additional stimulus associated with sterile inflammation, while cooperative activation by IL-3 and C5a may enhance BET-related antibacterial activity.

Monosodium urate (MSU) crystals can induce extracellular DNA release from isolated human basophils, leading to the formation of dense DNA-rich aggregates around the crystals within approximately two hours [67]. Basophils exposed to MSU crystals also showed increased expression of the activation marker cluster of differentiation 203c (CD203c), whereas no clear evidence of crystal internalization was observed [67]. This finding suggests that particle uptake was not required for the response [68]. These observations broaden the range of known BET inducers to include crystal-associated sterile inflammation [67]. However, the biological significance of these DNA aggregates remains unclear. Their presence in tissues or synovial fluid from patients with gout has not yet been convincingly demonstrated, and it therefore remains uncertain whether BETs contribute to the development or progression of human gout [69]. Together, these findings indicate that BET formation reflects the integration of priming-dependent and priming-independent signals arising in allergic, infectious, and sterile inflammatory settings (Figure 2).

### 4.2. NOX-Independent Oxidative Regulation of BET Formation

Unlike neutrophils, human basophils have only a limited capacity to generate ROS through the conventional phagocyte NADPH oxidase system [3]. Molecular analyses have failed to detect p47phox and p67phox, two cytosolic components required for the assembly of a functional NOX2 complex, in examined human basophils [3,70]. This molecular deficiency is consistent with their weak oxidative intracellular killing capacity and distinguishes them from professional phagocytes that depend on a robust respiratory burst for antimicrobial activity [3,5,70]. The ability of basophils to release ETs despite this limitation indicates that BET formation does not require canonical NOX2 activity [3]. Findings in other granulocytes similarly suggest that ET release can instead be supported by alternative sources of ROS, including mitochondria [3].

Although NOX2 activity is not required for BET formation, ROS still appear to be important for extracellular DNA release [3]. Diphenyleneiodonium (DPI) markedly reduced DNA externalization from activated human basophils, whereas stimulation through FcεRI or with C5a increased oxidant production in IL-3-primed cells [3,70]. However, because DPI inhibits several flavin-dependent enzymes, this finding does not identify the enzymatic source of the oxidant signal required for the response [3]. The increase in mtROS after basophil activation, together with the absence of essential NOX2 components, suggests that mitochondria are the more likely source [3]. The rapid externalization of mtDNA without immediate cell lysis also distinguishes BET formation from the slower, PMA-induced, NOX-dependent lytic pathway described in neutrophils [3,30]. Taken together, these findings suggest that BET formation is driven predominantly by mitochondrial oxidant production rather than NOX2 activity. However, the mitochondrial enzymes involved and the mechanisms linking oxidant generation to mtDNA release remain unclear.

### 4.3. Mitochondrial Regulation of BET Formation

Mitochondria appear to have a dual role in BET formation: they generate the oxidative signal required for the response and provide the mtDNA that forms the extracellular network [3,70]. In the most detailed study of human basophils to date, IL-3 alone did not induce trap release but primed the cells for mtDNA externalization following FcεRI-mediated activation, without an immediate loss of viability [3,23]. Inhibition of mtROS with mitochondria-targeted ubiquinone (MitoQ) markedly reduced extracellular mtDNA release, indicating that mtROS act upstream of DNA externalization rather than arising merely as a parallel consequence of basophil activation [3]. These findings support a sequence in which IL-3 increases basophil responsiveness, FcεRI activation stimulates mitochondrial oxidant production, and the resulting redox signal promotes mtDNA release [3,70]. However, the mechanisms through which mtROS initiate mtDNA mobilization across the mitochondrial membranes, through the cytoplasm, and ultimately into the extracellular space remain unclear.

Electron microscopy (EM) provides further evidence that BET release is centered on mitochondria and can occur without overt cell lysis [3]. Following FcεRI stimulation, mitochondria and cytoplasmic granules accumulated toward the leading region of activated human basophils, whereas nuclear architecture remained preserved [3,23]. The simultaneous detection of extracellular DNA and an intact nucleus argues against extensive decondensation of nuclear chromatin as the principal source of the released DNA and instead supports selective mtDNA externalization [2,3,5]. This response may allow basophils to contain microorganisms extracellularly without relying on phagocytosis or immediate destruction of the producing cell [5]. Preservation of cellular integrity also raises the possibility that basophils continue to release mediators or perform other immune functions after BET formation, although this has not been directly demonstrated. Because these conclusions derive largely from a single study and depend partly on experiments using pharmacological inhibitors, further investigation is needed to determine how mtDNA crosses mitochondrial membranes, moves through the cytoplasm, and exits the cell. The longer-term survival and functional activity of basophils following BET release also remain uncertain.

### 4.4. Established and Proposed Cellular Steps of Mitochondrial BET Release

The individual steps proposed to underlie BET formation are supported by different levels of experimental evidence. Direct studies in human basophils support IL-3-dependent priming, induction of extracellular DNA release through FcεRI or C5aR1, increased mitochondrial oxidant production, and mtROS-dependent mtDNA externalization [3]. They also show preservation of nuclear architecture during early BET release and redistribution of mitochondria and granules toward peripheral regions of activated cells [3]. By contrast, mitochondrial Ca^2+^ uptake, MCU-, mPTP-, and VDAC1-dependent membrane passage, nucleoid remodeling, intracellular mtDNA transport, and the final mechanism of plasma membrane crossing have not been demonstrated directly in basophils. These latter steps are discussed as testable mechanistic models derived from related immune-cell systems rather than as established components of BET formation.

#### 4.4.1. IL-3-Dependent Priming of Basophils

IL-3 acts as an early priming cytokine that increases the responsiveness of mature basophils to subsequent receptor stimulation, although it does not necessarily induce a complete effector response on its own [10]. It signals through a heterodimeric receptor composed of the IL-3-specific α-chain, cluster of differentiation 123 (CD123), and the common β-chain, cluster of differentiation 131 (CD131), which is also shared by the receptors for IL-5 and GM-CSF [10,71]. IL-3 binding primarily activates the Janus kinase 2–signal transducer and activator of transcription 5 (JAK2–STAT5) pathway, but also stimulates phosphoinositide 3-kinase–protein kinase B (PI3K–AKT) and rat sarcoma–RAF–MEK–ERK (RAS–RAF–MEK–ERK) signaling, induces proviral integration site for Moloney murine leukemia virus 1 (Pim1), and supports cell survival [10]. In primary human basophils, IL-3 induces rapid and concentration-dependent STAT5 phosphorylation, which reaches a peak approximately 10–20 min after stimulation, while producing little or no increase in the degranulation marker cluster of differentiation 63 (CD63) [72,73]. FcεRI activation produces a distinct response, characterized by a marked increase in CD63 expression but little detectable STAT5 phosphorylation [74]. When IL-3 and FcεRI stimulation are combined, degranulation remains intact, whereas STAT5 phosphorylation increases above the level induced by IL-3 alone [72]. This enhanced STAT5 response is reduced only when JAK2 and spleen tyrosine kinase (Syk) are inhibited simultaneously, suggesting that signals from the IL-3 receptor and FcεRI converge at the level of STAT5 regulation [72]. C5a-mediated activation provides another example of this priming effect. Brief exposure to IL-3 enhances C5a-induced histamine release and enables rapid leukotriene C4 (LTC4) production, whereas neither signal alone efficiently induces LTC4 synthesis [14,75]. Priming develops within minutes, varies according to IL-3 concentration and the order in which the stimuli are applied, and can remain effective for several hours [76]. In this setting, IL-3 induces phosphorylation of cytosolic phospholipase A2 (cPLA2) before C5a receptor activation, allowing the subsequent transient increase in intracellular Ca^2+^ to promote arachidonic acid release and leukotriene synthesis [14]. These findings suggest that IL-3 prepares selected signaling pathways and effector mechanisms before receptor activation rather than merely amplifying a response that is already underway. This may account for the requirement for IL-3 priming before certain stimuli can trigger BET formation. However, because these experiments did not directly measure extracellular mtDNA release, they do not demonstrate that STAT5, Pim1, or cPLA2 is directly involved in BET formation.

#### 4.4.2. FcεRI Activation and Downstream Signaling as the Second Signal for BET Formation

In primary human basophils, Morshed et al. demonstrated that FcεRI cross-linking with an anti-FcεRIα antibody induced concentration-dependent extracellular DNA release, but only in cells previously primed with IL-3 [3]. Neither IL-3 priming nor FcεRI stimulation alone elicited this response [3]. Under these conditions, approximately 60–80% of basophils formed extracellular DNA structures [3]. Furthermore, cross-linking of IgE-bound FcεRI activates an early Lyn–Syk tyrosine kinase pathway that has been directly demonstrated in primary human basophils [77]. Kepley et al. showed that receptor aggregation initially activates the Src-family kinase Lyn, followed by Syk and, to a lesser extent, zeta-chain-associated protein kinase 70 (ZAP-70), together with widespread phosphorylation of intracellular proteins [78]. Inhibition of Syk-family kinases reduced Syk and ZAP-70 activation, overall protein tyrosine phosphorylation, and IgE-dependent secretion without affecting Lyn activation, placing Lyn upstream of these kinases [78,79]. Other studies showed that disruption of Lyn activity delayed Syk phosphorylation, intracellular Ca^2+^ mobilization, and histamine release [80]. The importance of Syk is also evident in nonreleaser basophils, which lack detectable Syk and do not respond to FcεRI cross-linking [81]. When Syk expression is restored during culture with IL-3, IgE-dependent secretion also recovers [82]. These findings provide strong evidence that Lyn and Syk are central early components of FcεRI signaling in human basophils, although their involvement in BET formation has not been examined directly.

The steps connecting Syk activation to Ca^2+^ mobilization are less well defined in human basophils, and a complete signaling complex involving Syk, linker for activation of T cells (LAT), Src homology 2 domain-containing leukocyte protein of 76 kDa (SLP-76), and phospholipase C gamma (PLCγ) has not been directly demonstrated in these cells. Studies in mouse bone marrow-derived basophils (BMBs) show that SLP-76 is required for efficient FcεRI-dependent activation of phospholipase C gamma 2 (PLCγ2), degranulation, and IL-4 production. SLP-76 also contributes to PLCγ2 activation downstream of the IL-3 receptor, raising the possibility that priming and activating signals converge at this point [83,84]. Findings from mast cells suggest how the pathway may continue [79]. FcεRI aggregation induces phospholipase C gamma 1 (PLCγ1) phosphorylation and the hydrolysis of phosphatidylinositol 4,5-bisphosphate (PIP2) into inositol 1,4,5-trisphosphate (IP3) and diacylglycerol (DAG) [79]. IP3 releases Ca^2+^ from the endoplasmic reticulum (ER), whereas DAG acts together with Ca^2+^ to activate PKC [79]. This provides a plausible model involving adaptor proteins, PLCγ, IP3, and DAG, although the complete signaling sequence has not yet been confirmed in primary human basophils.

Primary human basophils nevertheless exhibit the principal Ca^2+^ response expected from this pathway [80,85]. FcεRI stimulation initially releases Ca^2+^ from intracellular stores, followed by sustained entry of extracellular Ca^2+^ [85]. The initial transient increase remains detectable when extracellular Ca^2+^ is chelated, whereas the sustained phase and full IgE-dependent degranulation are lost, demonstrating that release from intracellular stores alone is insufficient [80,85]. PI3K appears to strengthen the downstream response, as its inhibition reduces RAS–MEK–ERK activation, Ca^2+^ mobilization, histamine release, and LTC4 production without preventing Syk phosphorylation [86]. Collectively, the evidence supports a directly demonstrated Lyn–Syk pathway in human basophils, followed by a probable adaptor–PLCγ–Ca^2+^ sequence supported partly by findings from mouse basophils and mast cells. This pathway could connect FcεRI activation with changes in mitochondrial activity. However, it has not yet been demonstrated that PLCγ-dependent IP3 production, opening of IP3 receptors, or Ca^2+^ mobilization is required for mtROS generation or BET release.

#### 4.4.3. C5aR1-Mediated Activation of Primed Basophils

C5a provides an alternative activating signal for IL-3-primed basophils through C5aR1, also designated CD88, a seven-transmembrane G protein-coupled receptor expressed on human basophils [87]. Structural analyses have confirmed that activated C5aR1 can directly engage heterotrimeric Gi proteins, although these studies were not performed in primary basophils [88]. Its functional coupling to pertussis toxin-sensitive Gi/o proteins has, however, been demonstrated directly in human basophils [88,89]. Pretreatment with pertussis toxin almost completely suppressed mediator release induced by C5a, while responses to FcεRI cross-linking or direct calcium mobilization remained preserved [90]. These findings establish that C5aR1 and FcεRI initiate activation through different receptor-proximal mechanisms: C5aR1 signals through Gi/o proteins rather than through ITAM-dependent Lyn and Syk activation. Despite this distinction, both receptors ultimately influence calcium-dependent signaling, lipid mediator production, MAPK activation, and cellular metabolism, providing potential points of convergence downstream of their respective proximal pathways.

Following C5aR1 engagement, Gi/o activation is expected to release Gβγ subunits that stimulate PLCβ, most likely PLCβ2 or PLCβ3, and possibly PI3Kγ [91]. This arrangement is well established for chemoattractant receptors in related myeloid cells, but the individual PLCβ isoforms have not been directly examined in primary human basophils [91]. Available basophil studies nevertheless show that C5a induces a rapid and predominantly transient increase in cytosolic Ca^2+^, arising mainly from intracellular stores [92]. In unprimed cells, this short-lived signal is sufficient for substantial degranulation but does not efficiently support LTC_4_ synthesis [75]. IL-3 does not markedly increase the initial calcium peak; instead, it prepares downstream effector machinery so that the same transient C5a-induced signal can promote arachidonic acid release, leukotriene production, and more sustained functional activation [92]. The most plausible sequence therefore involves Gi/o-dependent activation of PLCβ, generation of IP_3_ and diacylglycerol, release of Ca^2+^ from intracellular stores, and subsequent engagement of PKC and MAPK pathways [93]. However, direct evidence linking a specific PLCβ isoform, IP_3_ receptor activation, or calcium mobilization to mtROS production and BET release is currently lacking. C5aR1 should therefore be regarded as an experimentally established BET-inducing receptor in primed basophils, whereas the intermediate molecular steps connecting receptor activation to mitochondrial responses remain inferred from conventional basophil signaling and related myeloid-cell models.

#### 4.4.4. Proposed Mitochondrial Ca^2+^–Metabolic Model Upstream of mtROS Generation

Receptor-induced cytosolic Ca^2+^ mobilization could provide a link between basophil activation and the mitochondrial oxidant response required for BET formation. One plausible model is that part of this cytosolic Ca^2+^ signal enters mitochondria through the mitochondrial calcium uniporter (MCU), alters mitochondrial metabolism, and increases electron flow through the respiratory chain, thereby favoring mtROS generation [3,94,95]. This sequence has not been demonstrated during BET formation. Basophil studies have neither measured mitochondrial matrix Ca^2+^ nor shown that MCU or its regulatory components are required for extracellular mtDNA release.

Support for such a connection comes from other granulocyte populations. FcεRI activation can increase mitochondrial Ca^2+^ uptake in mast cells, whereas altered regulation of the MCU complex in neutrophils has been linked to increased mitochondrial Ca^2+^ accumulation, superoxide production, and NET formation [94,95]. These findings show that receptor-driven Ca^2+^ signaling can influence mitochondrial redox activity in related immune cells, but they cannot establish the same pathway in basophils, particularly because the neutrophil model involved predominantly lytic NET formation.

What has been demonstrated directly in human basophils is narrower but important: FcεRI or C5aR1 activation of IL-3-primed cells increases mitochondrial oxidant production, and MitoQ reduces both this oxidant signal and extracellular mtDNA release [3]. Thus, mitochondrial oxidative activity lies upstream of BET-associated mtDNA externalization, whereas mitochondrial Ca^2+^ uptake, MCU activity, TCA-cycle stimulation, the responsible electron-transport-chain site, and mPTP opening remain proposed intermediate steps [96,97,98,99,100].

#### 4.4.5. mtROS as a Signaling Intermediate in BET Formation

Among the intracellular steps proposed for BET formation, the requirement for mitochondrial oxidative activity is supported most directly by basophil experiments. In IL-3-primed human basophils, receptor activation increases mitochondrial oxidant production, whereas MitoQ suppresses both the oxidant signal and extracellular mtDNA release [3]. In activated basophils, mtROS may oxidatively modify mitochondrial proteins and membrane lipids, alter membrane properties, reorganize mitochondrial nucleoids, and facilitate the mobilization and subsequent release of mtDNA [101,102]. They may also contribute to mitochondrial remodeling and redistribution toward the cell periphery [57]. However, none of these events has been directly demonstrated in BET-forming basophils, and the relevant redox-sensitive molecular targets remain unidentified. At controlled intracellular concentrations, ROS can function as signaling molecules in pathways involving PI3K–AKT–mechanistic target of rapamycin (mTOR) and mitogen-activated protein kinase (MAPK)–ERK signaling, although whether these pathways contribute to mtDNA release remains unknown [103,104]. Oxidants are also known to influence chromatin remodeling, granule enzyme redistribution, and membrane disruption during conventional lytic NETosis, but these predominantly nuclear processes should not be assumed to occur during the non-lytic release of mtDNA by basophils [105]. The available evidence therefore supports the conclusion that mitochondrial oxidative activity precedes and is required for extracellular mtDNA release. Its proposed effects on mitochondrial membranes, nucleoids, mitochondrial redistribution, and mtDNA transport remain to be tested directly.

#### 4.4.6. Proposed Nucleoid Remodeling and mtDNA Mobilization

Before mtDNA can become incorporated into the extracellular BET network, it must first be mobilized from mitochondrial nucleoids, compact DNA–protein structures in which the mitochondrial genome is organized primarily by mitochondrial transcription factor A (TFAM) and other nucleoid-associated proteins [106]. Because mtDNA is tightly packaged rather than freely dispersed within the mitochondrial matrix, its release is likely to require changes in nucleoid organization, such as reduced DNA compaction or partial disruption of nucleoid structure [106]. Mitochondrial oxidative signals may contribute to this process by modifying mtDNA, TFAM, or other nucleoid proteins, thereby altering DNA packaging and facilitating its mobilization [107,108]. However, such changes have not been directly visualized or biochemically characterized in activated basophils. It therefore remains unclear whether BETs contain intact circular mitochondrial genomes, shorter DNA fragments, TFAM-associated DNA, or a combination of these forms. It is also unknown whether mtDNA release is preceded by enlargement, relaxation, or oxidative modification of mitochondrial nucleoids. Findings from neutrophils support the possibility of an oxidant-dependent process. Following exposure to ribonucleoprotein (RNP)-containing ICs, neutrophils generated mitochondrial superoxide, partially lost their mitochondrial membrane potential, redistributed mitochondrial structures toward the cell periphery, and released extracellular material enriched in oxidized mtDNA [57]. Reducing mtROS decreased both DNA release and the associated interferon response, whereas oxidized DNA induced stronger signaling through stimulator of interferon genes (STING) than non-oxidized DNA [57]. These findings suggest that mtROS may influence not only mtDNA mobilization and release but also its molecular properties and immunostimulatory potential. Their relevance to BET formation remains uncertain, however, because it has not been established whether BET-derived mtDNA is oxidized, remains associated with TFAM, or activates TLR9, cyclic GMP–AMP synthase (cGAS)–STING, or other DNA-sensing pathways. Changes in mitochondrial nucleoid organization therefore remain a plausible but still unconfirmed step linking mtROS production to mtDNA release in basophils.

#### 4.4.7. Inferred Routes of mtDNA Passage Across Mitochondrial Membranes

How mtDNA crosses the inner and outer mitochondrial membranes during BET formation is unknown. No pore, transporter, or membrane-remodeling mechanism responsible for this step has been identified in basophils. A mechanistically relevant model comes from activated macrophages, in which MCU-dependent Ca^2+^ uptake, mPTP opening, and VDAC1 oligomerization have been linked to the movement of oxidatively processed mtDNA from the mitochondrial matrix into the cytosol [109,110]. Oxidatively damaged mtDNA was subsequently processed by DNA repair enzymes, including 8-oxoguanine DNA glycosylase 1 (OGG1) and flap endonuclease 1 (FEN1), generating fragments approximately 500–650 base pairs (bp) in length that were later detected in the cytosol [109]. However, the involvement of the mPTP does not necessarily indicate that large DNA fragments pass directly through a defined channel [109,111]. Instead, mPTP opening may destabilize the inner mitochondrial membrane, induce mitochondrial swelling, or alter contact sites between the inner and outer membranes, thereby facilitating the movement of mtDNA fragments toward oligomerized VDAC1 structures that may form larger openings in the outer membrane [109]. An alternative pathway involving BCL-2-associated X protein (BAX) and BCL-2 antagonist/killer 1 (BAK) macropores, together with herniation of the inner mitochondrial membrane, has been described during apoptosis [112]. However, its close association with apoptotic remodeling makes it a less likely explanation for rapid, non-lytic BET release. None of these potential routes has been directly examined in activated basophils, and the size and molecular form of mtDNA released during BET formation remain unknown. The proposed MCU–mPTP–VDAC1 sequence should therefore be regarded as a model derived from findings in another myeloid cell type rather than as an established pathway of mtDNA passage across mitochondrial membranes in basophils.

#### 4.4.8. Peripheral Mitochondrial Redistribution: Direct Observation and Proposed Transport Mechanisms

After crossing the mitochondrial membranes, mtDNA must be directed toward specific release sites at the cell surface rather than simply accumulating within the cytosol [3]. Observations in activated basophils suggest that this redistribution is spatially organized, as mitochondria and cytoplasmic granules accumulate at the leading edge of the cell, close to the sites from which thin extracellular DNA fibers emerge [3]. Studies of GM-CSF-primed, C5a-activated neutrophils provide one possible explanation for this process. In these cells, OPA1 preserved mitochondrial complex I activity and the availability of oxidized nicotinamide adenine dinucleotide (NAD^+^), thereby supporting the glycolytic production of adenosine triphosphate (ATP) required for continuous microtubule (MT) reorganization [113,114]. Loss of OPA1 activity prevented mitochondrial redistribution toward the plasma membrane and was accompanied by reduced MT formation, degranulation, and extracellular DNA release [113]. DNA externalization was also inhibited when MTs were either disrupted with nocodazole or excessively stabilized with taxol, indicating that dynamic MT reorganization, rather than their mere presence, is required for the response [113,115]. These findings support active, energy-dependent mitochondrial repositioning as part of ET formation, but they do not demonstrate that individual mitochondrial nucleoids are transported directly along MTs or establish the temporal sequence of mitochondrial movement, degranulation, and DNA release. In basophils, direct evidence is limited to the peripheral redistribution of mitochondria and cytoplasmic granules observed after activation [3]. The potential roles of OPA1, cellular metabolism, MTs, and motor proteins such as kinesins and dyneins have not yet been examined. It also remains unclear whether mtROS induce changes in the MT network or whether mitochondrial movement is regulated through a separate metabolic pathway. The proposed relationship among OPA1 activity, NAD^+^ availability, glycolytic ATP production, and MT reorganization therefore provides a useful model derived from neutrophil studies but should not yet be regarded as an established pathway of BET formation.

#### 4.4.9. Proposed Routes of mtDNA Passage Across the Plasma Membrane

The final membrane-crossing event remains unresolved. Basophil studies support rapid mtDNA externalization without evidence of widespread early plasma membrane rupture, but they do not reveal how DNA physically exits the cell [3]. Selective mtDNA release with preserved nuclear architecture supports a mitochondrial origin, whereas the maintenance of early cellular viability makes widespread plasma membrane rupture unlikely [3,41]. However, brief and highly localized membrane opening at the site of DNA extrusion cannot be excluded. In viable eosinophils, individual mtDNA release events have been described as catapult-like and occurring in less than one second, but these observations define only the rapid kinetics and spatial localization of DNA release rather than the mechanism through which mtDNA crosses the membrane [41,116]. One possibility is that mtDNA becomes enclosed within an intracellular membrane-bound compartment that transiently fuses with the plasma membrane and releases its contents at a defined site [117]. Direct fusion of an intact mitochondrion with the cell surface would be insufficient because mtDNA is located within the mitochondrial matrix and must first cross or become separated from the inner mitochondrial membrane [118]. Alternatively, mtDNA may leave the cell within extracellular vesicles (EVs) or larger mitochondria-derived membrane-bound structures and become exposed only after extracellular rupture or loss of their membrane coating [119]. The released DNA could then unfold and associate with basophil granule proteins. Although membrane-enclosed mitochondrial material has been reported in monocytes and other cell types, mtDNA-containing EVs and the cellular pathways required for their release have not been demonstrated in basophils [119]. Transient passage through a regulated protein pore represents another possibility, but GSDMD and related pore-forming proteins have not been examined during BET formation and are not required in every ET pathway [120]. Current evidence therefore supports a rapid and spatially restricted release process without clear evidence of widespread plasma membrane rupture [3]. Transient exocytosis, release within EVs, and passage through regulated pores remain plausible mechanisms, but each requires direct imaging and experimental validation in basophils.

#### 4.4.10. Extracellular Assembly and Functional Maturation of BETs

Once mtDNA has crossed the plasma membrane, the released DNA and basophil granule products must assemble into an organized extracellular BET network [3]. Findings from vital eosinophil extracellular trap (EET) formation suggest that DNA and granule proteins are not necessarily released as a preassembled complex [116]. In cytokine-primed eosinophils, degranulation and extracellular EPX release reach near-maximal levels within approximately one minute, whereas extracellular mtDNA continues to accumulate and peaks later [116]. This difference in timing suggests that the two components may be released separately and associate only after entering the extracellular space [116]. Comparable kinetic studies have not yet been performed in basophils. Human BETs contain mtDNA together with basogranulin, whereas murine BETs contain mtDNA and mouse mast cell protease 8 (mMCP-8), but these observations demonstrate only that the components colocalize within the same extracellular regions [3]. They do not establish which component is released first or whether these proteins bind directly to DNA [3]. Granule proteins may associate with negatively charged DNA through electrostatic and other non-covalent interactions, although the binding strength, relative proportions, and molecular basis of basogranulin or mMCP-8 association with mtDNA remain unknown [116]. The physical form of the released mtDNA is also unclear. After externalization, the DNA and granule products appear to spread and assemble into an extended fibrous network.

**Figure 3:**
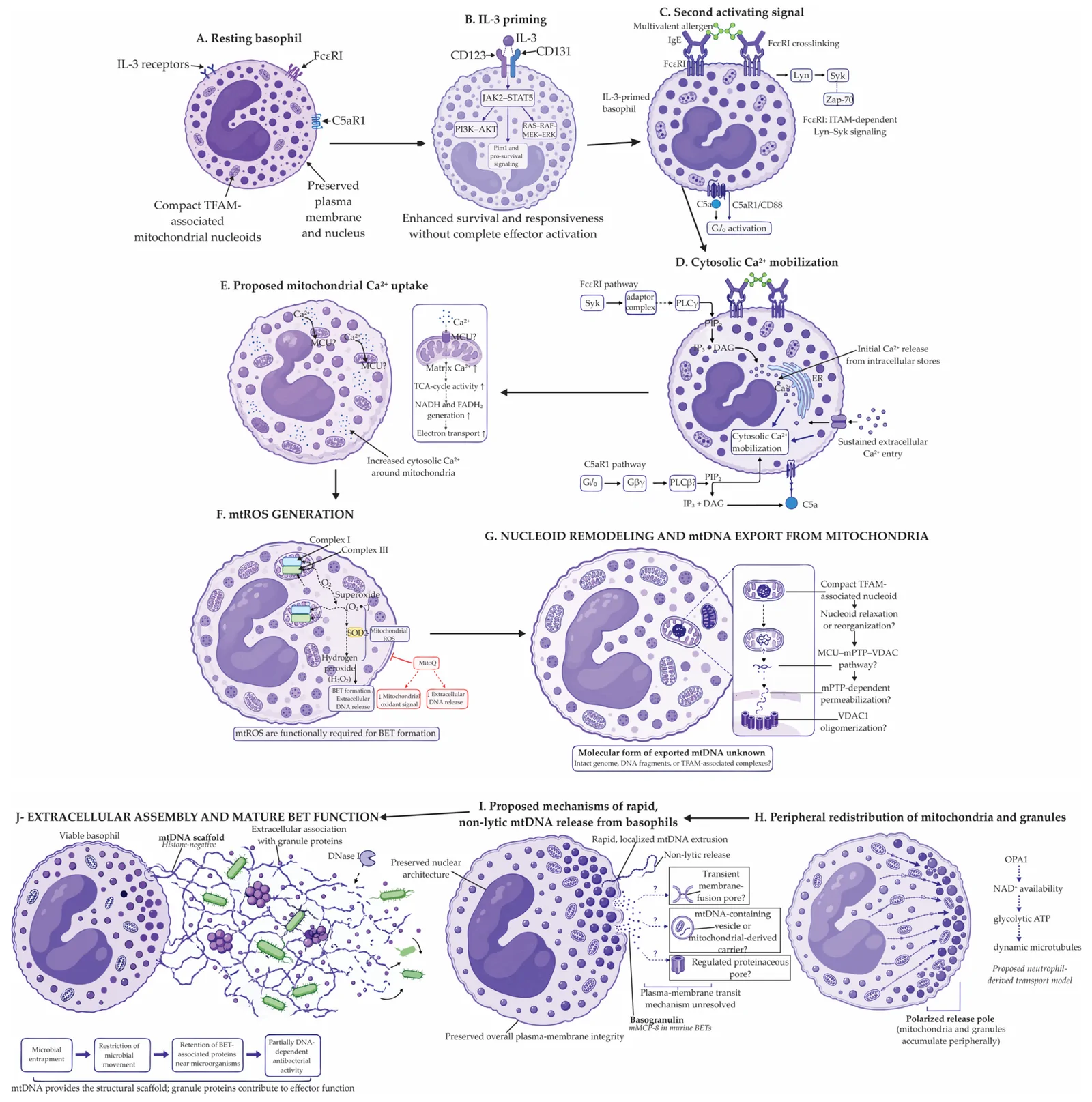
Evidence-supported and proposed cellular events involved in mitochondrial DNA release and BET formation. Direct studies in basophils support IL-3-dependent priming, activation through FcεRI or C5aR1, cytosolic Ca^2+^ mobilization, increased mitochondrial oxidant production, mtROS-dependent extracellular mtDNA release, peripheral redistribution of mitochondria and granules, association of extracellular mtDNA with basophil granule proteins, and BET-mediated microbial entrapment and antibacterial activity. The molecular events connecting these experimentally supported observations remain incompletely defined. Mitochondrial Ca^2+^ uptake and MCU involvement are proposed on the basis of findings obtained mainly in mast cells and neutrophils; nucleoid remodeling and oxidant-dependent mtDNA mobilization are inferred primarily from neutrophil and other mitochondrial DNA-release models; the MCU–mPTP–VDAC1 mechanism of mtDNA passage across mitochondrial membranes derives predominantly from macrophage studies; and OPA1- and microtubule-dependent mitochondrial transport is based largely on neutrophil experiments. Likewise, the mechanism by which mtDNA crosses the basophil plasma membrane remains unknown, with rapid release, vesicular transport, transient membrane fusion, and regulated pore formation representing possibilities derived from eosinophils and other cell systems rather than mechanisms demonstrated in basophils.

The resulting mtDNA network helps organize basophil antimicrobial activity by trapping microorganisms and retaining BET-associated proteins close to their surfaces [5]. Human basophils can reduce the viability of *Escherichia coli* and *Staphylococcus aureus* despite their limited phagocytic activity [5]. Deoxyribonuclease I (DNase I) only partially reduces this antibacterial effect, suggesting that extracellular DNA contributes to microbial control but acts together with soluble mediators or other DNA-independent processes [5]. Although basogranulin and mMCP-8 have been detected in human and murine BETs, respectively, their individual contributions to bacterial killing have not been determined [3]. The broader protein composition of BETs also remains poorly characterized. Their antibacterial activity therefore most likely reflects the combined effects of the extracellular mtDNA network and its associated basophil-derived products rather than the activity of mtDNA or any single granule protein alone. Overall, current evidence supports a core BET pathway in which IL-3 priming and receptor-dependent activation are followed by increased mitochondrial oxidant production and extracellular mtDNA release. The intervening events that may connect receptor signaling to mitochondrial activation, mtDNA mobilization, membrane passage, intracellular transport, and extracellular assembly remain unevenly defined. The mitochondrial Ca^2+^/MCU, nucleoid-remodeling, MCU–mPTP–VDAC1, microtubule-transport, and plasma membrane export models discussed above should therefore be viewed as experimentally testable hypotheses rather than consecutive steps of an established BET pathway (Figure 3).

### 4.5. DNA and Protein Components of BETs

In the experimental models, the extracellular DNA within BETs is derived predominantly from mitochondria rather than the nucleus [3]. This conclusion is supported by the selective reduction in intracellular mitochondrial cytochrome c oxidase subunit 1 (COX1) DNA sequences following basophil activation, whereas nuclear 18S ribosomal DNA (18S rDNA) remains unchanged [3]. Histones are also absent from the extracellular fibers and remain confined within the intact nucleus, arguing against direct externalization of nuclear chromatin [3]. Because most intracellular mtDNA remains detectable after activation, BET formation appears to involve the selective release of only a fraction of the mitochondrial DNA pool rather than the complete mtDNA content of the cell [3]. BETs therefore differ from lytic nuclear ETs in both the origin of their DNA and the mechanism through which the extracellular network is generated [3]. Nevertheless, the mitochondrial origin of the released DNA should be verified in each experimental setting rather than inferred solely from the presence of extracellular DNA near basophils.

The extracellular mtDNA network also contains basophil granule-derived proteins, indicating that BETs are multicomponent structures. Confocal microscopy has demonstrated colocalization of extracellular DNA with basogranulin in human basophils and with mMCP-8 in murine basophils [3]. These proteins may help confirm the basophil origin of the structures, but colocalization alone does not demonstrate direct binding to mtDNA or define the strength, stoichiometry, or molecular nature of these interactions. The complete protein composition of BETs has not yet been established, and the mature network may also contain mitochondrial, cytoplasmic, membrane-derived, or extracellular proteins. BETs are therefore most appropriately described as extracellular mtDNA networks associated with basophil granule products, whose composition may differ between species and vary according to the activating stimulus and surrounding tissue environment.

#### Inflammatory Signaling Induced by Mitochondrial DNA

Once released into the extracellular space, mtDNA can act as a damage-associated molecular pattern (DAMP) because it retains several features of bacterial DNA, including a relatively high abundance of poorly methylated cytosine–phosphate–guanine (CpG) motifs [121,122]. Following cellular uptake, one of the best-established routes of mtDNA recognition involves TLR9, which detects DNA within acidified endolysosomal compartments rather than at the cell surface [123,124]. DNase I and deoxyribonuclease 1-like 3 (DNASE1L3) can process extracellular DNA before its cellular uptake, whereas deoxyribonuclease II (DNase II) continues DNA degradation within endolysosomes [125]. This processing may generate shorter mtDNA fragments that are more readily recognized by TLR9 [126]. Receptor activation induces dimerization and MyD88 recruitment, followed by nuclear factor kappa B (NF-κB)-dependent expression of inflammatory genes [123]. In responsive cells, trafficking of TLR9 to specialized lysosome-related compartments can also activate interferon regulatory factor 7 (IRF7) and promote IFN-I production [123]. When mtDNA is enclosed within EVs, endocytosis may deliver it directly to endosomal TLR9 or allow it to reach the cytosol after release from the vesicle [127]. Its intracellular destination is therefore an important determinant of the resulting response. DNA retained within endosomes can activate TLR9, whereas cytosolic mtDNA can be recognized by cGAS–STING or absent in melanoma 2 (AIM2) and may also contribute indirectly to activation of the NLR family pyrin domain containing 3 (NLRP3) inflammasome [121]. Oxidative damage may enhance the immunostimulatory activity of mtDNA, but no specific sensing pathway should be assumed unless DNA uptake and delivery to the relevant intracellular compartment have been demonstrated [128].

The effects of mtDNA recognition depend strongly on its mode of release, site of accumulation, and access to specific sensing pathways. Studies of intracellular mtDNA release during infection show that cytosolic mtDNA can activate cGAS–STING-associated autophagy and IFN-I signaling, as well as inflammasome responses that may contribute to microbial restriction [122,129]. However, mtDNA itself has not been shown to exert direct bactericidal activity [130]. Depending on the pathogen and cellular environment, mtDNA-induced immune responses may support microbial control or instead promote excessive inflammation and tissue injury [122,129]. Outside the context of infection, extracellular and circulating mtDNA may contribute to local or systemic sterile inflammation, particularly when it is oxidized or inefficiently cleared [131]. Experimental administration of mtDNA has induced arthritis-like inflammation, whereas elevated levels of cell-free mtDNA have been reported in rheumatoid arthritis and in conditions associated with aging, cellular senescence, defective mitophagy, and systemic IFN-I activity [132,133]. These findings may be relevant to BETs because their extracellular DNA is predominantly mitochondrial and may retain its immunostimulatory potential after release. However, it remains unknown whether BET-associated mtDNA is oxidized, internalized by neighboring cells, or capable of activating TLR9, cGAS–STING, AIM2, or inflammasome-related pathways. BET-derived mtDNA should therefore be considered potentially immunostimulatory, although the receptors involved and the resulting inflammatory effects require direct experimental investigation.

## 5. Biological Roles of BETs in Host Defense and Inflammation

Experimental studies indicate that BETs can contribute directly to extracellular bacterial control, whereas their detection in helminth-associated inflammation and human inflammatory skin diseases demonstrates that these structures can also form within complex tissue environments in vivo [3,5]. These two lines of evidence should be interpreted separately. Antibacterial activity has been evaluated directly, whereas the presence of BETs within tissues does not establish whether they are protective, harmful, or merely associated with local basophil activation [5]. Their biological effects are likely to vary according to the initiating stimulus, the composition of immune and stromal cells within the tissue, and the persistence and clearance of extracellular mtDNA and its associated proteins [134]. In some settings, BET formation may help restrict microbial dissemination, whereas in others it may simply reflect basophil activation or contribute to the amplification of ongoing inflammation [3,5]. Because the strength and nature of the available evidence differ substantially among disease settings, the detection of BETs within a lesion should not by itself be considered evidence of either host protection or tissue injury.

The low abundance of basophils places an important limit on how the biological contribution of BETs should be interpreted. In inflammatory settings dominated by neutrophils, particularly acute bacterial infection, basophils are unlikely to generate an extracellular-trap burden comparable to that produced by the much larger neutrophil population. No study has yet quantified BET and NET formation side by side within the same lesion or infection model, however, and their relative contribution cannot currently be assigned on a quantitative basis. Cell number may also be less informative in tissues in which basophils are selectively recruited. Repeated helminth challenge, for example, produced marked local basophil accumulation, with extracellular traps detected in more than 30% of the basophils present in the affected skin [3]. Similarly, basogranulin-associated extracellular DNA has been detected in a substantial fraction of tissue basophils in several inflammatory skin diseases [3]. These observations suggest that BETs are better viewed as a spatially restricted and context-dependent effector mechanism than as a major source of extracellular traps across the inflammatory compartment. During bacterial inflammation dominated by neutrophils, BETs are unlikely to represent a major source of extracellular traps compared with NETs. By contrast, in allergic or type 2 inflammatory settings where basophils preferentially accumulate, the local release of mtDNA and granule-derived proteins may exert biologically meaningful effects despite the overall scarcity of basophils in the circulation and tissues. Whether this localized activity measurably alters pathogen control, inflammatory amplification, or tissue injury remains unknown.

### 5.1. Antibacterial Functions of Basophil Extracellular Traps

BET formation provides basophils with a direct extracellular mechanism for controlling microorganisms, complementing their better-established role in regulating immune responses [1,5]. The DNA network can trap microorganisms, restrict their movement through surrounding tissues, and retain them in close proximity to effector molecules released by basophils [5,134]. This mechanism may be particularly important because basophils have limited phagocytic capacity compared with professional phagocytes and therefore cannot rely to the same extent on intracellular microbial uptake and killing [1]. By releasing BETs, basophils can participate in innate defense without internalizing bacteria, although the importance of this response during naturally occurring infections remains unclear [5].

Antibacterial activity against *Escherichia coli* and *Staphylococcus aureus* has been demonstrated in primary human basophils and homeobox B8 (Hoxb8)-derived murine basophils [5]. Confocal microscopy showed fluorescently labeled bacteria trapped within extracellular DNA networks, whereas live-cell imaging revealed nonviable bacteria associated with these structures [2,5]. Treatment with DNase I released the trapped microorganisms and partially reduced bacterial killing, indicating that the DNA network contributes to both bacterial retention and loss of viability but does not fully account for either effect [5]. Soluble mediators and basophil-derived proteins associated with the DNA are therefore also likely to contribute. The extracellular fibers were clearly separated from the Hoechst-positive nucleus, supporting a non-nuclear origin of the released DNA [5]. The cells also excluded propidium iodide (PI), consistent with DNA release occurring without immediate loss of plasma membrane integrity [5].

Direct bacterial exposure produced measurable antibacterial activity, and this response was enhanced when basophils were first primed with IL-3 and subsequently stimulated with C5a [3,5]. These findings suggest that bacteria can initiate the extracellular response directly, whereas cytokine and complement signals increase basophil responsiveness and strengthen bacterial control [3,5]. The individual proteins responsible for damaging bacteria have not yet been identified, and it remains uncertain whether microbial killing or physical containment represents the more important contribution of BETs in vivo. Only a small number of bacterial species have been examined, and the range of microorganisms susceptible to BET-associated containment or killing remains poorly defined. More importantly, no study has directly compared the relative contribution of BETs and NETs under the same infectious conditions. The relative contribution of BET formation to neutrophil-rich antimicrobial responses therefore remains unclear. Current evidence suggests that BETs may have a more limited, locally relevant role, rather than a contribution comparable to the much better established NET response. [2].

### 5.2. BET Formation During Helminth Infection

Basophils are recruited into tissues during helminth infection, where they participate in type 2 immune responses and local antiparasitic defense [135]. BET formation in vivo has been investigated in mice using an intradermal model of *Nippostrongylus brasiliensis* infection that induces pronounced basophil accumulation within the skin [3]. Repeated challenge resulted in substantially greater basophil recruitment than a single exposure, and ETs were detected in more than 30% of the basophils present within repeatedly challenged lesions [3]. The colocalization of extracellular DNA with the murine basophil granule marker mMCP-8 supported the basophil origin of these structures [3]. These findings demonstrate that BETs can form within parasite-induced inflammatory tissues and are not restricted to experiments performed ex vivo or in vitro [3]. Their presence suggests that extracellular DNA release forms part of the basophil response to helminth-associated inflammation. However, it remains unclear whether BETs directly trap or damage the parasite, influence the recruitment or activity of other immune cells, or merely reflect prolonged basophil activation within the affected tissue.

### 5.3. Basophil Extracellular Traps in Inflammatory Skin Diseases

Bullous pemphigoid (BP), Wells syndrome (WS), eosinophilic folliculitis (EF), atopic dermatitis (AD), and urticaria differ substantially in their clinical manifestations and underlying pathogenic mechanisms [136]. Nevertheless, all of these disorders may involve inflammatory environments in which type 2 mediators and recruited granulocytes contribute to the development or persistence of cutaneous lesions [137]. Basophils may participate in these responses by infiltrating affected skin and releasing histamine, lipid mediators, cytokines, and granule proteins that influence vascular permeability, leukocyte recruitment, and local immune activation [13,137]. In BP, basophils accumulate within an inflammatory environment rich in autoantibodies and granulocytes and associated with separation of the epidermis from the dermis [138]. WS and EF are characterized by prominent eosinophilic infiltrates, within which basophils may provide an additional source of type 2 mediators [136]. Basophils are generally less abundant in AD and urticaria, but their number may not fully reflect their biological influence because even a small population can remain highly responsive within tissues rich in cytokines, chemokines, or IgE [139]. Their detection across these disorders suggests recurrent involvement in cutaneous inflammation, although their contribution is likely to differ among diseases and may include initiating the response, sustaining established inflammation, or being recruited after the inflammatory process has already developed [139].

BET formation has also been directly observed within these inflammatory skin lesions [3]. The structures were identified by the colocalization of extracellular DNA with basogranulin and appeared as long, thin fibers extending from individual basophils into areas containing neighboring inflammatory cells [3]. BETs were detected in BP, WS, EF, AD, and urticaria, with basogranulin-associated extracellular DNA present in approximately 15–40% of the tissue basophils examined [3]. The finding that only a subset of basophils formed BETs suggests that the response depends on local activating signals and is restricted to particular cells within the inflammatory infiltrate [3,137]. Comparable structures were not detected in normal skin, in which basophils were only occasionally observed, indicating that tissue recruitment alone is probably insufficient and that additional inflammatory stimulation is required [3,139]. The extracellular fibers may help retain basophil-derived material within densely inflamed regions, but their biological effects in these lesions remain unclear. Their presence does not establish whether BETs contribute to epithelial or vascular injury, prolong eosinophil-rich inflammation, or promote chronic tissue damage. It also remains uncertain whether they actively participate in disease pathogenesis or merely indicate intense local basophil activation. A balanced synthesis of the available evidence, its principal limitations, and the potential clinical relevance of BETs across infectious and inflammatory settings is provided in Table 1.

## 6. Basophil Extracellular Traps in Cancer

Basophils are increasingly recognized as context-dependent participants in tumor immunity whose effects can vary substantially with the surrounding cellular and molecular environment [13]. In some settings, they may support angiogenesis, tissue remodeling, and immunosuppressive macrophage activity, whereas in others they may contribute to the recruitment and activation of cytotoxic lymphocytes [140,141]. BET formation could represent an additional, still largely unexplored mechanism through which basophils influence the tumor microenvironment (TME) [3,142]. At present, this possibility is supported mainly by parallels with ETs released by other granulocytes and by preliminary reports of circulating basophil-associated traps in patients with cervical cancer [6]. However, neither line of evidence demonstrates that BETs directly promote tumor progression or contribute to antitumor immunity. For this reason, the cancer-related evidence is best considered at three distinct levels: effects demonstrated for basophils within tumors, observations directly involving BET-like structures in patients with cancer, and mechanisms inferred from other extracellular-trap systems that remain untested in basophils.

### 6.1. Context-Dependent Roles of Basophils in Cancer

Basophils can influence tumor development indirectly by modifying the behavior of other cells within the TME [13]. In the lung, resident basophils contribute to the regulation of macrophage development and can promote an M2-like phenotype [19]. This provides one possible explanation for how basophils might influence macrophage states associated with tissue remodeling and immune suppression in tumors [143]. Basophil activity has also been associated with increased tumor growth in experimental models of inflammation-driven skin carcinogenesis [13,144]. Although the underlying mechanisms were not fully defined, they may involve the release of cytokines, histamine, lipid mediators, and angiogenic factors, together with changes in neighboring immune and stromal cells [143,144]. These findings should not be generalized to all cancers because the effects of basophil activation are likely to depend on the tumor type and the surrounding inflammatory and cellular environment.

In other experimental models, basophils appear to support antitumor responses rather than tumor growth [145]. In melanoma, their activity has been associated with stronger chemotactic signaling, increased infiltration of cluster of differentiation 8-positive (CD8^+^) T cells, and more effective cytotoxic responses during tumor rejection [141]. These findings demonstrate that basophils can exert markedly different and sometimes opposing effects depending on the tumor context. However, most available studies have focused on mediator release and interactions with other cell populations [141]. There is currently no direct evidence that BET formation drives macrophage polarization, angiogenesis, tumor-associated inflammation, or CD8^+^ T-cell recruitment in these models. The protumor and antitumor effects described here therefore represent relevant biological settings in which the specific contribution of BETs remains to be investigated.

These opposing observations are particularly relevant when considering where BET formation might fit within the TME. Basophils do not act as isolated effector cells; their influence depends on the immune populations that surround them and on the cytokine environment that determines their activation state. Associations between peripheral basophils and M2 macrophage infiltration in advanced gastric cancer, together with experimental evidence linking IL-4-dependent signaling to protumorigenic myelopoiesis, point toward settings in which basophil-derived mediators could reinforce an immunoregulatory myeloid compartment. Conversely, basophil-dependent chemotactic recruitment of CD8^+^ T cells and IL-3-driven T cell–basophil crosstalk demonstrate that the same cell population can participate in immune circuits that favor tumor control. BET release could theoretically modify either type of interaction by retaining mtDNA and granule-derived products within a restricted extracellular space, but there is currently no evidence that BET formation is required for macrophage polarization, T-cell recruitment, or either of these opposing tumor outcomes. This distinction is important: the established biology of tumor-associated basophils defines the cellular setting in which BETs may operate, but it does not yet establish BETs as mediators of that biology.

### 6.2. Tumor-Specific Roles of Basophils

Basophil activity in cancer varies considerably between tumor types and appears to be shaped by the local immune context in which these cells are recruited and activated [145]. Their effects may depend on the composition of the immune infiltrate, the cytokine milieu, the anatomical site, and the stage of disease [143,146]. In some tumors, basophil recruitment or activation has been associated with stronger cytotoxic immune responses and more favorable clinical features, whereas in others it has been linked to Th2-dominant inflammation, accumulation of immunoregulatory myeloid cells, or more aggressive disease [141,145]. Differences between circulating and tumor-infiltrating basophils further suggest that peripheral blood counts do not necessarily reflect their activity within the tumor microenvironment. Studies in melanoma, lung cancer, colorectal cancer, and other malignancies have begun to define the specific settings in which basophils may favor antitumor immunity or, conversely, contribute to tumor progression.

- Melanoma

Melanoma provides one of the clearest examples of the functional plasticity of tumor-associated basophils [145]. Earlier experimental work showed that basophils can support tumor rejection rather than tumor growth. In murine HCmel12 and B16 melanoma models, profound regulatory T-cell depletion was followed by basophil accumulation that preceded CD8^+^ T-cell infiltration [141]. Tumor-associated basophils produced CCL3 and CCL4, and basophil depletion impaired CD8^+^ T-cell recruitment and prevented efficient tumor rejection [141]. Increasing basophil numbers with IL-3/anti-IL-3 complexes likewise enhanced the recruitment of tumor-specific CD8^+^ T cells and improved tumor control [141]. More recent studies have identified an additional IL-3-dependent antitumor mechanism. In B16 melanoma, progressive CTL exhaustion was accompanied by declining IL-3 production; restoration of IL-3 increased intratumoral basophils, while IL-3-activated basophils improved CTL survival and IFN-γ production through basophil-derived IL-4 [145]. Basophil depletion weakened the antitumor effect of IL-3, whereas adoptively transferred activated basophils slowed tumor growth and prolonged survival. IL-3 supplementation also reduced experimental melanoma metastasis [145]. These findings establish two mechanistically distinct settings in which basophils can reinforce cytotoxic antitumor immunity.

Human melanoma, however, reveals the opposite side of this biology. Tajima et al. recently identified BB1-positive basophils in primary cutaneous melanoma and sentinel lymph nodes (SLNs) [147]. Higher basophil infiltration in primary lesions was associated with greater Breslow depth and shorter progression-free survival [147]. Basophils were also present in metastasis-negative SLNs at early disease stages, where their abundance increased from stage I to stage II and correlated with IL-4 expression; in situ hybridization confirmed IL4 production by the infiltrating basophils [147]. The authors proposed that basophil-derived IL-4 may help establish a Th2-polarized environment before overt nodal metastasis [147]. Thus, even within melanoma, basophil-derived IL-4 cannot be assigned a fixed biological meaning: in an experimentally generated CTL-dominant environment it can sustain cytotoxic T-cell function, whereas in human sentinel nodes it may contribute to a Th2-biased milieu associated with progression.

- Lung cancer

Lung cancer provides another setting in which basophils may influence tumor biology through mechanisms that extend beyond the immediate tumor compartment. In human and murine non-small-cell lung cancer, IL-4 derived from bone marrow basophils and eosinophils was shown to act on granulocyte–monocyte progenitors and promote the development of immunosuppressive, tumor-supporting myeloid cells [143]. Basophil depletion in the corresponding experimental models reduced tumor burden and partially normalized myelopoiesis, supporting a functional contribution of these cells rather than a simple association [143].

A more direct interaction between human basophils and lung adenocarcinoma cells has been demonstrated in vitro. Schroeder et al. showed that coculture of basophils with A549 cells produced a contact-dependent response involving IL-3 priming, basophil-bound IgE, and A549-associated galectin-3 [148]. Basophils contributed IL-4 and IL-13, whereas the coculture induced A549-derived IL-6 and VEGF-A; notably, IL-3-primed basophils retained the ability to induce IL-6 and VEGF-A from A549 cells even after fixation, supporting an important contribution of cell-surface interactions [148]. These findings identify a bidirectional basophil–tumor-cell interaction capable of generating inflammatory and angiogenic mediators. However, the experiments were performed in vitro and did not assess extracellular DNA release. They therefore provide evidence for basophil–lung cancer crosstalk, but not for BET formation or a BET-dependent tumor-promoting mechanism.

- Colorectal cancer

Recent colorectal cancer data make the importance of anatomical compartment even clearer. Rahkola et al. examined tumor tissue from two independent CRC cohorts comprising 1830 patients and quantified basophils by double immunohistochemistry using proMBP1 together with cytokeratin. Higher intratumoral basophil density was associated with lower tumor stage, less frequent lymphovascular invasion, and lower cancer-specific mortality. After adjustment for major prognostic variables, including stage and mismatch-repair status, the hazard ratio for cancer-specific mortality comparing high with low basophil density was 0.67 in the first cohort and 0.54 in the second, with the latter association clearly significant. Circulating basophil counts correlated only weakly with tumor basophil density and were not themselves associated with prognosis in that study [149]. These findings provide some of the strongest current human evidence that locally recruited basophils can mark a favorable immune context within a solid tumor.

At the same time, a separate analysis of pretreatment peripheral immune composition in stage I–III CRC reached a different conclusion for circulating cells. Richards et al. found that higher circulating basophil counts and proportions were associated with worse overall survival, with stronger associations in patients with rectal tumors and earlier-stage disease [150]. These findings do not necessarily conflict with the results of the larger tissue-based study. Basophils detected within tumors represent cells that have already migrated into and interacted with the local microenvironment, whereas circulating basophil counts are influenced by recruitment from the blood as well as by systemic inflammatory responses. Peripheral counts may therefore differ substantially from both the abundance and the functional state of basophils within the tumor. Earlier clinical studies have likewise produced variable associations between peripheral basophil levels and CRC outcome [151]. The recent CRC studies therefore highlight the importance of distinguishing between circulating basophils and those present within the tumor tissue [149]. Future BET studies should follow the same principle, distinguishing circulating BET-like material from extracellular traps demonstrated spatially within tumor tissue rather than treating the two as interchangeable biomarkers. Neither of the recent CRC studies assessed extracellular trap formation.

- Other tumor settings

Comparable context dependence is evident in other malignancies. In advanced gastric cancer, higher baseline peripheral basophil counts were associated with a lower response rate and shorter progression-free and overall survival in patients receiving anti-PD-1 therapy combined with chemotherapy [140]. Tumor-infiltrating basophils were spatially associated with M2 macrophages, and the abundance of basophils and M2 macrophages was greater in nonresponders, supporting a possible link between basophils and an immunoregulatory myeloid environment [140]. Pancreatic ductal adenocarcinoma provides an established mechanistic example of a tumor-promoting basophil circuit: IL-4-producing basophils accumulate in tumor-draining lymph nodes, correlate with the intratumoral Th2/Th1 balance and poorer postoperative survival, and are recruited through a pathway involving CCL7-producing alternatively activated monocytes and activated by T-cell-derived IL-3. Basophil-deficient mice also showed impaired long-term pancreatic tumor establishment, supporting a functional rather than purely correlative role [146]. By contrast, ovarian cancer studies have associated higher circulating basophil numbers, greater ex vivo basophil responsiveness, and transcriptional evidence of activated intratumoral basophils with better patient survival [152]. More recently, a study of clear-cell renal cell carcinoma found that higher preoperative circulating basophil counts were independently associated with high WHO/ISUP grade and perirenal or renal-sinus fat invasion, again illustrating that the prognostic direction of basophil-associated signals differs among tumor types [153].

### 6.3. Potential Role of BETs in the Tumor Microenvironment

ETs are increasingly being investigated as active components of the TME rather than merely as by-products of inflammation [154,155,156]. Most available evidence concerns NETs, which have been associated with tumor progression, metastatic dissemination, reactivation of dormant cancer cells, vascular obstruction, and cancer-associated thrombosis [157,158]. These findings raise the possibility that ETs released by other leukocyte populations may also influence tumor behavior. Cancer cells may induce not only lytic release of nDNA but also rapid, non-lytic release of mtDNA from viable immune cells [159,160]. In one experimental model, anaplastic thyroid cancer cells induced mtDNA-based ET formation in neutrophils, demonstrating that signals derived from malignant cells can activate this type of response [159]. This observation provides a relevant comparison for BETs. However, there is currently no direct evidence that tumor cells induce non-lytic mtDNA release from basophils or that the same signaling and membrane-transport mechanisms are involved.

- Potential Tumor-Promoting Consequences of BET Formation

Several mechanisms could potentially link BET formation to tumor-promoting inflammation, although direct evidence in cancer is currently lacking. Persistent extracellular mtDNA may contribute to the damage-associated signals generated within chronically inflamed tumors [161,162]. In contrast to many NETs, which predominantly contain nuclear chromatin, the best-characterized BETs contain mainly mtDNA. This difference suggests that, if BETs influence the tumor microenvironment, their inflammatory activity may be more closely related to recognition of extracellular or internalized DNA than to the toxic effects of extracellular histones. It remains unknown whether BET-derived mtDNA is taken up by tumor, stromal, or myeloid cells and gains access to TLR9, cGAS–STING, AIM2, or other inflammasome-associated sensing pathways. Evidence obtained with cell-free mtDNA does not establish that DNA released within BETs behaves in the same way.

Second, the extracellular network may increase the local persistence of basophil-derived mediators [3]. Basophils can release histamine, lipid mediators, cytokines, proteases, and angiogenic factors, and their association with extracellular DNA could alter the spatial distribution or duration of these signals within the TME [13]. Such retention could, in principle, influence vascular permeability, macrophage state, stromal remodeling, or tumor-cell behavior without requiring BETs to exert direct cytotoxic effects. This possibility is particularly relevant in tumors in which basophil activity is already associated with angiogenic or immunoregulatory programs.

Third, parallels with NET biology raise the possibility that extracellular DNA networks might affect tumor-cell adhesion, migration, metastatic seeding, or vascular complications [163]. NETs can contribute to pre-metastatic niche formation, suppression of CD8^+^ T-cell activity, metastatic behavior, angiogenesis, and cancer-associated thrombosis [154,164]. These findings demonstrate what extracellular DNA–protein networks are capable of doing within cancer-associated inflammation, but they do not establish that BETs reproduce these effects. The lower abundance of basophils, the mitochondrial origin of BET DNA, and their distinct protein cargo make direct extrapolation particularly uncertain. To establish a role for BETs in cancer, it will not be sufficient simply to detect them morphologically in tumor tissue. Their localization within the tumor microenvironment should be demonstrated, and experimental studies should show that altering BET formation changes a relevant tumor-associated response.

- Potential Contribution of BETs to Antitumor Immunity

The possibility that BETs contribute to antitumor immunity deserves equal consideration. Basophils can support tumor rejection through mechanisms that include chemotactic recruitment of CD8^+^ T cells, and more recent experimental work has identified IL-3-dependent crosstalk between T cells and basophils as a pathway that can strengthen antitumor responses [145]. These observations show that basophil activation within cancer is not inherently tumor promoting. Whether extracellular trap release participates in these responses, however, remains unknown.

One possibility is that localized BET formation could retain inflammatory or granule-derived mediators close to activated immune cells and thereby reinforce short-range communication within the TME. Extracellular DNA networks might also restrict the dispersal of cellular material released during tumor injury or treatment and influence its uptake by antigen-presenting cells. Conversely, prolonged exposure to extracellular mtDNA could drive chronic innate signaling that ultimately favors immune dysfunction rather than tumor rejection. The direction of the response may therefore depend on the amount and persistence of BET material, the cells exposed to it, and the inflammatory state of the tumor. At present, there is no evidence that BET formation enhances antigen presentation, CD8^+^ T-cell priming, cytotoxicity, or tumor rejection directly. These possibilities should therefore be tested independently from the established antitumor effects of basophils themselves.

### 6.4. Circulating BET-like Structures in Cervical Cancer

Cervical cancer (CC) develops within tissues affected by persistent virus-driven epithelial transformation, local inflammation, vascular remodeling, and, during treatment, additional tissue injury [165]. Neutrophils and other myeloid populations have been studied more extensively in this setting, whereas the contribution of basophils remains poorly understood, and direct evidence linking them to CC progression is limited [166,167]. Because basophils release vasoactive and immunoregulatory mediators, they could influence systemic inflammation or local immune responses within the tumor [142]. A small clinical study reported extracellular trap-like structures in peripheral blood smears from some patients receiving combined anticancer therapy [6]. The structures were considered to originate from basophils because of their morphology, but their composition was not examined in sufficient detail to confirm that they were true BETs. In particular, mitochondrial DNA content, colocalization with basophil-specific granule proteins, preservation of basophil viability, and rigorous exclusion of extracellular traps released by other granulocyte populations were not comprehensively demonstrated. The structures were also detected in peripheral blood rather than within cervical tumor tissue. They are therefore more appropriately referred to as BET-like structures. Their presence suggests that systemic granulocyte activation may occur during combined anticancer treatment, but it does not establish intratumoral BET formation or a direct influence on malignant cells or the cervical tumor microenvironment.

The reported frequency of ETs varied among the small treatment groups. Following radiotherapy (RT) combined with ftorafur, NETs were detected in approximately half of the examined patients, generally as only one or two structures per smear, whereas individual samples contained between one and seven structures interpreted as BET-like [6]. Among patients treated with RT and cisplatin, NETs were detected in 37.5% of cases, with two to fifteen structures per smear, whereas BET-like structures were reported in 12.5%, with one to nine structures per smear [6]. Because the treatment subgroups were small and the structures were identified in peripheral blood smears, these findings should be regarded as preliminary observations rather than reliable evidence of differences between therapeutic regimens. The concurrent presence of NETs and BET-like structures in some patients suggests that treatment may generate a systemic inflammatory environment capable of activating multiple granulocyte populations, but it does not demonstrate that the same pathway induces both responses. Tissue injury and inflammatory signals generated during treatment may contribute to their formation, although the responsible triggers were not examined directly [6]. The available evidence therefore associates combined therapy with the appearance of circulating BET-like structures but does not establish the mechanisms or stimuli responsible for their formation. It also remains unknown whether comparable structures are present within cervical tumors or whether circulating or intratumoral BETs influence vascular inflammation, tumor-cell behavior, metastatic dissemination, or antitumor immunity.

### 6.5. Clinical and Translational Relevance of BETs in Cancer

The clinical relevance of BETs in cancer remains undefined, but several questions can now be formulated in testable terms. The first is whether circulating BETs reflect tumor-associated basophil activation, treatment-induced tissue injury, or a broader systemic inflammatory response. The limited cervical cancer observations cannot distinguish among these possibilities because BET-like structures were evaluated in peripheral blood rather than tumor tissue and were not correlated with treatment response, toxicity, progression, or survival [6]. Consequently, circulating BETs should not currently be regarded as biomarkers of tumor burden or therapeutic efficacy.

A second question is whether BET formation within tumors reflects local basophil activity more accurately than peripheral blood basophil counts. This distinction may be important because basophils are numerically rare and their peripheral abundance may not reflect selective recruitment or activation within individual tumors. Studies of tumor tissue should confirm that extracellular DNA is associated with basophil-specific proteins and is predominantly mitochondrial in origin. At the same time, the spatial relationship of these structures to tumor cells, other immune cells, endothelial cells, and stromal cells should be documented. Such approaches would make it possible to determine whether BETs preferentially occur in necrotic areas, vascular niches, invasive fronts, immune aggregates, or treatment-damaged regions.

Therapeutic targeting of BETs requires even stronger evidence. Strategies developed against NETs, including nuclease-mediated degradation of extracellular DNA or inhibition of upstream trap-forming pathways, cannot simply be transferred to BETs. BET formation may coexist with beneficial basophil functions, including support of cytotoxic immune-cell recruitment, and extracellular DNA degradation could also remove potentially protective microbial or inflammatory containment mechanisms. A BET-directed therapeutic strategy would therefore require evidence that these structures actively contribute to tumor progression or treatment-related toxicity, together with an intervention that can modify BET formation without broadly suppressing basophil function.

Before BETs can be considered as therapeutic targets, their presence, biological activity, and contribution to tumor behavior need to be established in relevant cancer models and human tissues. Prospective studies should determine whether BETs are present within tumor tissue, whether their abundance changes during systemic therapy or radiotherapy, and whether they correlate with defined immune states, vascular complications, metastatic behavior, or clinical outcome. Until such data are available, BETs are best considered a candidate marker and mechanistic component of tumor-associated basophil activation rather than an established prognostic factor or therapeutic target.

**Figure 4:**
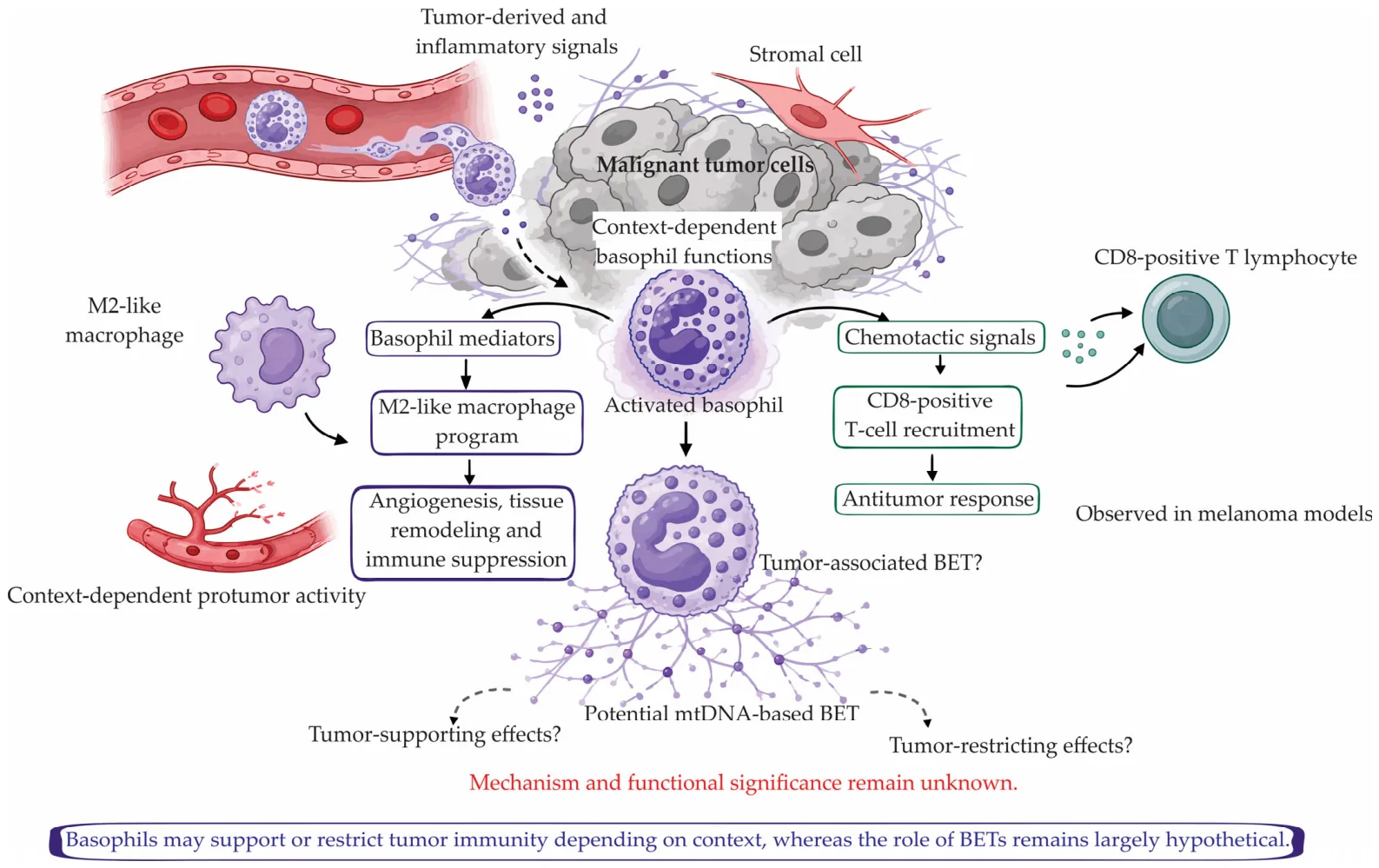
Context-dependent roles of basophils and the hypothetical contribution of basophil extracellular traps (BETs) in cancer. Tumor-derived and inflammatory signals may recruit and activate basophils within the tumor microenvironment, where their conventional functions can exert either tumor-promoting or tumor-restricting effects depending on the cellular and cytokine context. Experimentally supported basophil activities include the release of mediators capable of promoting M2-like macrophage polarization, angiogenesis, tissue remodeling, and immunosuppression, as well as the production of chemotactic signals associated with CD8-positive T-cell recruitment and antitumor responses, particularly in experimental melanoma models. Experimentally supported basophil-mediated pathways contributing to these tumor-promoting or tumor-restricting effects are indicated by solid arrows. In contrast, the formation and functional consequences of tumor-associated BETs remain largely unresolved. A potential mtDNA-based BET phenotype is illustrated as a conceptual model, but neither direct induction of BET formation by tumor cells nor BET-mediated tumor-promoting or tumor-restricting activity has been established. Hypothetical or currently unconfirmed relationships involving tumor-associated BET formation and potential BET-mediated tumor-supporting or tumor-restricting effects are indicated by dashed arrows.

Taken together, the current evidence positions BETs as a plausible but still unproven link between basophil activation, tumor-associated inflammation, and treatment-related systemic responses, as schematically illustrated in Figure 4 and summarized in Table 2.

## 7. Conclusions

Basophil extracellular traps represent a distinct addition to the effector repertoire of basophils, with the best-characterized response involving rapid release of mitochondrial DNA, preservation of nuclear architecture and early plasma membrane integrity, dependence on mitochondrial oxidant activity, and association of the extracellular DNA network with basophil-derived granule proteins. Although these features distinguish BET formation from classical lytic NETosis, they are not entirely unique to basophils and overlap in part with mitochondrial trap formation described in other granulocytes. The strongest functional evidence for BETs remains their ability to entrap bacteria and contribute to extracellular antimicrobial activity, while their detection in helminth-associated inflammation and human inflammatory skin lesions confirms that BET formation also occurs in vivo. At the same time, the low abundance of basophils suggests that BETs are more likely to act as a localized and context-dependent effector mechanism than as a quantitatively dominant source of extracellular traps. Their broader inflammatory, tissue-damaging, vascular, and tumor-related effects remain insufficiently defined, and current evidence does not justify considering BETs an established biomarker or therapeutic target. The key unresolved question is therefore no longer whether basophils can form extracellular traps, but how much this response contributes to host defense and disease within complex inflammatory environments where other, more abundant trap-forming cells are simultaneously active.

## Figures and Tables

**Table 1:** Context-dependent biological roles and potential clinical relevance of basophil extracellular traps in host defense and inflammation.

Biological Setting	Evidence Base	BET-Related Finding	Clinical Significance/ Therapeutic Implication	Key Take-Home Message	References
Extracellular antibacterial defense	Primary human basophils and Hoxb8-derived murine basophils exposed to *Escherichia coli* or *Staphylococcus aureus*	BET networks retained bacteria and were associated with reduced bacterial viability. DNase I released trapped microorganisms but only partly reduced killing, indicating that extracellular DNA acts together with basophil-derived proteins and soluble mediators.	BETs may provide basophils with a means of limiting bacterial spread despite their relatively weak phagocytic capacity. Therapeutic degradation of extracellular DNA would therefore need to balance the reduction in harmful extracellular material against a possible loss of microbial containment.	Antibacterial activity is the most directly demonstrated biological function of BETs, although its importance during naturally occurring infections remains uncertain.	[1,2,5,134]
Helminth-associated inflammation	Intradermal *Nippostrongylus brasiliensis* infection in mice, particularly after repeated challenge	Extracellular DNA colocalized with mMCP-8 in basophil-rich skin lesions, and traps were detected in more than 30% of recruited basophils after repeated exposure.	These findings establish that BETs can form in vivo during parasite-driven inflammation. However, there is not yet sufficient evidence to consider BETs an antiparasitic effector mechanism or a therapeutic target in helminth infection.	Tissue detection confirms BET formation but does not show that the traps directly immobilize or damage the parasite.	[3,135]
Inflammatory skin diseases	Human tissue samples from bullous pemphigoid, Wells syndrome, eosinophilic folliculitis, atopic dermatitis, and urticaria	Basogranulin-associated extracellular DNA was detected in approximately 15–40% of tissue basophils, whereas comparable structures were not identified in normal skin.	BETs may serve as indicators of strong local basophil activation and could retain inflammatory material within lesions. Their value as biomarkers or therapeutic targets remains uncertain because a causal contribution to tissue injury or disease persistence has not been established.	BET formation is reproducibly associated with inflamed skin, but its protective, pathogenic, or bystander role remains unresolved.	[3,13,136,137,138,139]

**Table 2:** Current evidence and clinical relevance of basophil extracellular traps in cancer.

Cancer-Related Setting	Model/Sample Type	Detection/Assessment Approach	Main Finding and Relevance to BETs	Strength of Evidence	Clinical Significance/ Major Limitation
Basophils and immunosuppressive myeloid responses in gastric cancer	Human advanced gastric cancer; peripheral blood and primary tumor samples from patients treated with anti-PD-1 plus chemotherapy or chemotherapy alone	Peripheral blood basophil counts; immunohistochemistry and immunofluorescence for tumor-infiltrating basophils and M2 macrophages; correlation with treatment response and survival	Higher peripheral basophil counts were associated with greater intratumoral basophil and M2 macrophage infiltration and poorer outcome during anti-PD-1 plus chemotherapy. BET formation was not investigated.	Moderate clinical evidence for basophil association; no direct BET evidence	Supports a relationship between basophils and an immunoregulatory TME, but does not establish that basophils cause M2 polarization or that BETs participate in treatment resistance.
Basophils in inflammation-driven skin tumor growth	Murine inflammation-driven skin carcinogenesis, including DMBA–TPA models and genetically modified mice affecting IgE/FcεRI signaling or basophil abundance	Flow cytometry, tissue immunostaining, qRT-PCR, genetic loss-of-function models, basophil depletion, histamine/FcεRI pathway analysis, and tumor-growth assessment	IgE-bearing basophils accumulated in inflamed and tumor-associated skin, and FcεRI-dependent basophil activity promoted epithelial proliferation and tumor outgrowth, partly through histamine. BETs were not examined.	Strong experimental evidence for a protumor basophil function; no direct BET evidence	Demonstrates that activated basophils can promote tumor development, but this effect cannot currently be attributed to extracellular trap formation.
Basophil-derived IL-4 and protumorigenic myelopoiesis	Human and murine non-small-cell lung cancer; tumor tissue and murine genetic models	Single-cell RNA sequencing, conditional IL-4Rα deletion, basophil depletion, analysis of bone marrow progenitors and tumor-infiltrating myeloid cells	Basophil- and eosinophil-derived IL-4 contributed to programming of immunosuppressive tumor-promoting myeloid cells; basophil depletion reduced tumor burden. BET formation was not assessed.	Strong mechanistic evidence for basophil involvement; no direct BET evidence	Establishes a systemic route by which basophils can shape the TME through bone marrow myelopoiesis, but provides no evidence that BETs mediate this effect.
Basophils and CD8^+^ T-cell recruitment during tumor rejection	Murine melanoma models, including HCmel12 and B16-based tumors, under conditions of regulatory T-cell depletion or experimentally induced basophilia	Flow cytometry, basophil and CD8^+^ T-cell infiltration analysis, chemokine blockade, IL-3-induced basophilia, adoptive T-cell transfer, in vitro migration assays, and tumor-growth assessment	Intratumoral basophils promoted CCL3/CCL4-dependent CD8^+^ T-cell recruitment and enhanced tumor rejection. No extracellular traps were measured.	Strong experimental evidence for an antitumor basophil function; no direct BET evidence	Provides direct evidence that basophils can favor tumor control, emphasizing that basophil activation cannot be regarded as uniformly protumorigenic.
IL-3-driven T cell–basophil crosstalk and antitumor immunity	Murine B16-OVA melanoma and E.G7-OVA tumor models; isolated T cells and basophils	Flow cytometry, recombinant or tumor-derived IL-3 supplementation, engineered IL-3-expressing T cells/tumor cells, basophil depletion and adoptive transfer, cytokine assays, tumor-growth and survival analyses	CTL-derived IL-3 activated basophils, which in turn enhanced CTL activity and restricted tumor growth. BET release was not evaluated.	Strong experimental evidence for basophil-dependent antitumor immunity; no direct BET evidence	Identifies an antitumor immune circuit in which basophils are functionally required, but whether BET formation contributes to this interaction remains unknown.
Potential tumor-promoting activity of BETs within the TME	No direct BET cancer model; hypothesis derived from BET composition, extracellular mtDNA biology, tumor-associated basophil studies, and NET cancer models	No BET-specific detection. Mechanistic inference from extracellular mtDNA signaling and experimental studies of NETs involving tumor progression, immune suppression, angiogenesis, metastasis, and thrombosis	Persistent BET-associated mtDNA and granule proteins could theoretically amplify local inflammatory signaling, alter macrophage or endothelial behavior, or affect tumor-cell adhesion, migration, angiogenesis, metastatic seeding, or vascular complications. None of these effects has been demonstrated directly for BETs.	Indirect/hypothesis-generating evidence	A protumor role is biologically plausible, but extrapolation from NETs is limited by the different DNA origin, protein composition, oxidative machinery, and abundance of basophils.
Potential contribution of BETs to antitumor immunity	No direct BET cancer model; hypothesis derived from experimentally demonstrated antitumor functions of basophils	No BET-specific detection. Functional inference from basophil-dependent CD8^+^ T-cell recruitment and IL-3-driven T cell–basophil interactions	Local BET formation could theoretically retain basophil-derived mediators or modify short-range immune-cell interactions, but BETs have not been shown to enhance antigen presentation, T-cell priming, cytotoxicity, or tumor rejection.	Indirect/hypothesis-generating evidence	Beneficial basophil functions caution against indiscriminate inhibition of BET formation before its role in antitumor immunity is defined.
Circulating BET-like structures in cervical cancer	Human peripheral blood from 28 patients with stage IIb–III cervical cancer undergoing radiotherapy alone or radiotherapy combined with ftorafur or cisplatin	Peripheral blood smear analysis; hematoxylin–eosin staining and light microscopy at ×1400 magnification; morphological enumeration of extracellular traps	BET-like structures were reported in a small proportion of patients, particularly following combined treatment. They were detected in peripheral blood rather than tumor tissue and were identified morphologically without molecular confirmation of basophil origin, mtDNA, or BET-associated proteins.	Direct but very low-strength BET evidence: preliminary morphological observation	This is the only direct clinical report of cancer-associated BET-like structures, but it cannot establish bona fide BET formation, intratumoral occurrence, biological function, prognostic value, or treatment specificity.
Clinical and translational relevance of BETs in cancer	No validated prospective clinical cohort or tumor-tissue study specifically assessing BETs	No standardized BET assay. Future studies require combined extracellular-DNA visualization, basophil-specific markers, confirmation of mitochondrial DNA and associated proteins, and spatial tissue analysis	It remains unknown whether circulating and intratumoral BETs represent the same biological process, whether tumors themselves induce BET formation, and whether BET abundance correlates with immune phenotype, progression, metastasis, treatment response, toxicity, or survival.	Insufficient evidence for clinical application	Current evidence does not support BETs as biomarkers or therapeutic targets. The immediate priority is biological and methodological validation in tumor tissue and longitudinal clinical cohorts.
Direct basophil–tumor-cell crosstalk in lung adenocarcinoma	Human peripheral blood basophils and culture-derived basophils cocultured with A549 human lung adenocarcinoma cells	Basophil–A549 coculture; IL-3 priming; IgE sensitization; galectin-3-deficient A549 clones; cell-contact experiments; cytokine measurement; fixed versus viable basophils	Contact-dependent basophil–A549 interaction involved IL-3 priming, basophil-associated IgE, and A549 galectin-3. Basophils produced IL-4/IL-13, while A549 cells produced IL-6 and VEGF-A. Extracellular DNA release and BET formation were not assessed.	Direct in vitro evidence for basophil–tumor-cell crosstalk; no direct BET evidence	Demonstrates reciprocal signaling between human basophils and lung adenocarcinoma cells with inflammatory and angiogenic consequences, but its relevance in vivo and any contribution of BETs remain unknown.

## Data Availability

As this is a review article, no new data were created or analyzed. All data supporting the findings of this study are available in the cited literature. Therefore, a data availability statement is not applicable.

## Abbreviations

The following abbreviations are used in this manuscript:

AD	atopic dermatitis
AIM2	absent in melanoma 2
AKT	protein kinase B
AMPs	antimicrobial peptides
Ang-1	angiopoietin-1
AREG	amphiregulin
ATP	adenosine triphosphate
BAK	B-cell lymphoma 2 antagonist/killer 1
BAX	B-cell lymphoma 2-associated X protein
BETs	basophil extracellular traps
BMBs	bone marrow-derived basophils
BP	bullous pemphigoid
bp	base pairs
C5a	complement component 5a
C5aR1	complement C5a receptor 1
CC	cervical cancer
CCL2	C-C motif chemokine ligand 2
CCL5	C-C motif chemokine ligand 5
CCL7	C-C motif chemokine ligand 7
CCL8	C-C motif chemokine ligand 8
CCL11	C-C motif chemokine ligand 11
CCL13	C-C motif chemokine ligand 13
CCR2	C-C chemokine receptor 2
CCR3	C-C chemokine receptor 3
CD8^+^	cluster of differentiation 8-positive
CD63	cluster of differentiation 63
CD123	cluster of differentiation 123
CD131	cluster of differentiation 131
CD203c	cluster of differentiation 203c
cGAS	cyclic guanosine monophosphate–adenosine monophosphate synthase
COX1	cytochrome c oxidase subunit 1
CpG	cytosine–phosphate–guanine
cPLA2	cytosolic phospholipase A2
CysLTs	cysteinyl leukotrienes
DAG	diacylglycerol
DAMP	damage-associated molecular pattern
DNA	deoxyribonucleic acid
DNASE1L3	deoxyribonuclease 1-like 3
DNase I	deoxyribonuclease I
DNase II	deoxyribonuclease II
DNases	deoxyribonucleases
DPI	diphenyleneiodonium
ECP	eosinophil cationic protein
EDN	eosinophil-derived neurotoxin
EETs	eosinophil extracellular traps
EF	eosinophilic folliculitis
EM	electron microscopy
EoP	eosinophil progenitor
EPX	eosinophil peroxidase
ER	endoplasmic reticulum
ERK	extracellular signal-regulated kinase
ETC	electron transport chain
ETosis	extracellular trap-associated cell death
ETs	extracellular traps
EVs	extracellular vesicles
FADH_2_	reduced flavin adenine dinucleotide
FcεRI	high-affinity receptor for immunoglobulin E
FEN1	flap endonuclease 1
fMLF	N-formyl-methionyl-leucyl-phenylalanine
GM-CSF	granulocyte–macrophage colony-stimulating factor
GSDMD	gasdermin D
GTPase	guanosine triphosphatase
GzmB	granzyme B
HGF	hepatocyte growth factor
Hoxb8	homeobox B8
ICs	immune complexes
IFN-I	type I interferons
IgE	immunoglobulin E
IL-3	interleukin-3
IL-4	interleukin-4
IL-5	interleukin-5
IL-13	interleukin-13
IP_3_	inositol 1,4,5-trisphosphate
IRF7	interferon regulatory factor 7
ITAM	immunoreceptor tyrosine-based activation motif
JAK2	Janus kinase 2
LAT	linker for activation of T cells
LPS	lipopolysaccharide
LTA	lipoteichoic acid
LTC_4_	leukotriene C4
Lyn	Lck/Yes-related novel tyrosine kinase
MAPK	mitogen-activated protein kinase
MBP	major basic protein
MCU	mitochondrial calcium uniporter
MEK	mitogen-activated protein kinase kinase
MICU1	mitochondrial calcium uptake 1
MitoQ	mitochondria-targeted ubiquinone
mMCP-8	mouse mast cell protease 8
MPO	myeloperoxidase
mPTP	mitochondrial permeability transition pore
MSU	monosodium urate
MT	microtubule
mtDNA	mitochondrial DNA
mTOR	mechanistic target of rapamycin
mtROS	mitochondrial reactive oxygen species
MyD88	myeloid differentiation primary response 88
NAD^+^	oxidized nicotinamide adenine dinucleotide
NADH	reduced nicotinamide adenine dinucleotide
NADPH	nicotinamide adenine dinucleotide phosphate
nDNA	nuclear DNA
NE	neutrophil elastase
NETosis	neutrophil extracellular trap formation
NETs	neutrophil extracellular traps
NF-κB	nuclear factor kappa B
NLRP3	NLR family pyrin domain containing 3
NOX	NADPH oxidase
NOX2	NADPH oxidase 2
OGG1	8-oxoguanine DNA glycosylase 1
OPA1	optic atrophy 1
p47phox	47 kDa phagocyte oxidase subunit
p67phox	67 kDa phagocyte oxidase subunit
PAD4	peptidylarginine deiminase 4
PAF	platelet-activating factor
pDCs	plasmacytoid dendritic cells
PI	propidium iodide
PI3K	phosphoinositide 3-kinase
Pim1	proviral integration site for Moloney murine leukemia virus 1
PIP_2_	phosphatidylinositol 4,5-bisphosphate
PKC	protein kinase C
PLCγ	phospholipase C gamma
PLCγ1	phospholipase C gamma 1
PLCγ2	phospholipase C gamma 2
PMA	phorbol 12-myristate 13-acetate
RAF	rapidly accelerated fibrosarcoma kinase
RAS	rat sarcoma GTPase
rDNA	ribosomal DNA
RNP	ribonucleoprotein
ROS	reactive oxygen species
RT	radiotherapy
SIRT1	sirtuin 1
SK	small-conductance calcium-activated potassium channels
SLP-76	Src homology 2 domain-containing leukocyte protein of 76 kDa
SOD2	superoxide dismutase 2
Src	proto-oncogene tyrosine-protein kinase Src
STAT5	signal transducer and activator of transcription 5
STING	stimulator of interferon genes
Syk	spleen tyrosine kinase
TANs	tumor-associated neutrophils
TCA	tricarboxylic acid
TFAM	mitochondrial transcription factor A
Th2	T helper 2
TLR2	Toll-like receptor 2
TLR4	Toll-like receptor 4
TLR9	Toll-like receptor 9
TLRs	Toll-like receptors
TME	tumor microenvironment
TSLP	thymic stromal lymphopoietin
VDAC1	voltage-dependent anion channel 1
VEGF-A	vascular endothelial growth factor A
WS	Wells syndrome
ZAP-70	zeta-chain-associated protein kinase 70
